# Potential crosstalk between ferroptosis and immunosenescence in osteoarthritis: evidence integration and translational insights from the osteoimmune microenvironment

**DOI:** 10.3389/fimmu.2026.1824999

**Published:** 2026-07-08

**Authors:** Jinglin Li, Fuyin Yang, Xuan Deng, Lin Zhang, Xianpeng Huang, Yang Yu, Huazhang Xiong

**Affiliations:** 1 Department of Orthopedics, Affiliated Hospital of Zunyi Medical University, Zunyi, China; 2 Joint Orthopaedic Research Center of Zunyi Medical University, University of Rochester Medical Center, Zunyi, China; 3 Department of Orthopedics Center, Renhuai People’s Hospital, Zunyi, China

**Keywords:** ferroptosis, immunosenescence, iron homeostasis, lipid peroxidation, osteoarthritis, osteoimmune microenvironment

## Abstract

Osteoarthritis (OA) has traditionally been regarded as a degenerative disease primarily characterized by cartilage wear and tear. However, accumulating evidence suggests that it is fundamentally a whole-joint disorder involving the coordinated participation of cartilage, synovium, subchondral bone, and immune components. In recent years, ferroptosis and immunosenescence have each been recognized as contributors to OA initiation and progression, yet their potential interplay within the osteoimmune microenvironment remains insufficiently integrated. This review summarizes how iron homeostasis imbalance, lipid peroxidation, and impaired antioxidant defense promote ferroptosis in joint-resident cells, and how immunosenescence influences joint homeostasis through chronic low-grade inflammation and functional remodeling. It further analyzes the possible crosstalk between these two processes in cartilage, synovium, subchondral bone, and related immune cells. In addition, this review outlines current advances in therapeutic strategies, including anti-ferroptotic interventions, anti-senescence modulation, and optimization of local delivery approaches. At present, direct evidence supporting a stable causal loop between ferroptosis and immunosenescence in OA remains limited, and many of the proposed mechanisms are still largely derived from *in vitro* studies, animal models, and extrapolation from other disease contexts. Therefore, this review aims to provide a testable working framework for understanding the link between ferroptosis and immunosenescence in OA from the perspective of the osteoimmune microenvironment and evidence stratification, and to offer reference for the development of future mechanism-oriented therapeutic strategies.

## 1 Introduction

OA is one of the most common chronic degenerative joint diseases and a major cause of pain, limited mobility, and reduced quality of life in middle-aged and elderly individuals (1). Traditionally, OA has been regarded as a degenerative disorder primarily driven by articular cartilage wear and mechanical injury. However, increasing evidence indicates that OA is not merely a localized cartilage lesion, but rather a whole-joint disease involving cartilage, synovium, subchondral bone, and surrounding immune components (2, 3). In addition to abnormal mechanical loading, processes such as chronic low-grade inflammation, metabolic imbalance, oxidative stress, and aging-related alterations are also widely involved in OA initiation and progression, collectively shaping a persistently dysregulated pathological microenvironment within the joint (4). With the deepening of research in this field, increasing attention has been directed toward the role of the osteoimmune microenvironment in OA progression. Chondrocytes, synovial cells, bone cells, and infiltrating immune cells within the joint do not exist in isolation; rather, they continuously engage in signaling exchange and functional coupling within a complex local microenvironment. Once the homeostasis of this microenvironment is disrupted, inflammatory amplification, impaired tissue repair, and structural degeneration may reinforce one another, thereby driving persistent disease progression (5, 6). In this context, ferroptosis and immunosenescence, two biological processes closely associated with aging, inflammation, and loss of tissue homeostasis, have gradually been recognized as potentially important contributors to OA pathology.

Ferroptosis is a form of regulated cell death characterized by iron-dependent lipid peroxidation accumulation and membrane damage (7). Existing studies have shown that iron homeostasis imbalance, enhanced lipid peroxidation, and impaired antioxidant defense systems can promote ferroptosis in chondrocytes and other joint-related cells, thereby aggravating matrix degradation, inflammatory responses, and tissue injury (8). On the other hand, immunosenescence, as a major manifestation of organismal aging, can weaken the capacity of joint tissues to maintain homeostasis through immune cell functional remodeling, persistent chronic low-grade inflammation, and abnormal release of inflammatory mediators, thus creating conditions favorable for the chronic progression of OA (9). Notably, abnormalities in iron metabolism, oxidative stress, and inflammatory microenvironment remodeling may constitute a shared basis linking ferroptosis and immunosenescence, suggesting that these two processes in OA may not be independent, but rather potentially interconnected (10, 11). Nevertheless, there is still a lack of systematic integration regarding how ferroptosis and immunosenescence interact within the osteoimmune microenvironment of OA, which cell populations are primarily involved, and to what extent this relationship is specific to OA. Although current evidence suggests that these two processes may form mutually reinforcing interactions within the joint microenvironment, most studies are still based on *in vitro* experiments, animal models, and associative analyses, and many key mechanisms and causal relationships remain to be further validated. Therefore, a comprehensive synthesis of ferroptosis and immunosenescence from the perspective of the osteoimmune microenvironment may not only deepen our understanding of the complex pathogenesis of OA, but also provide new insights for the identification of mechanism-oriented therapeutic strategies.

**Figure 1:**
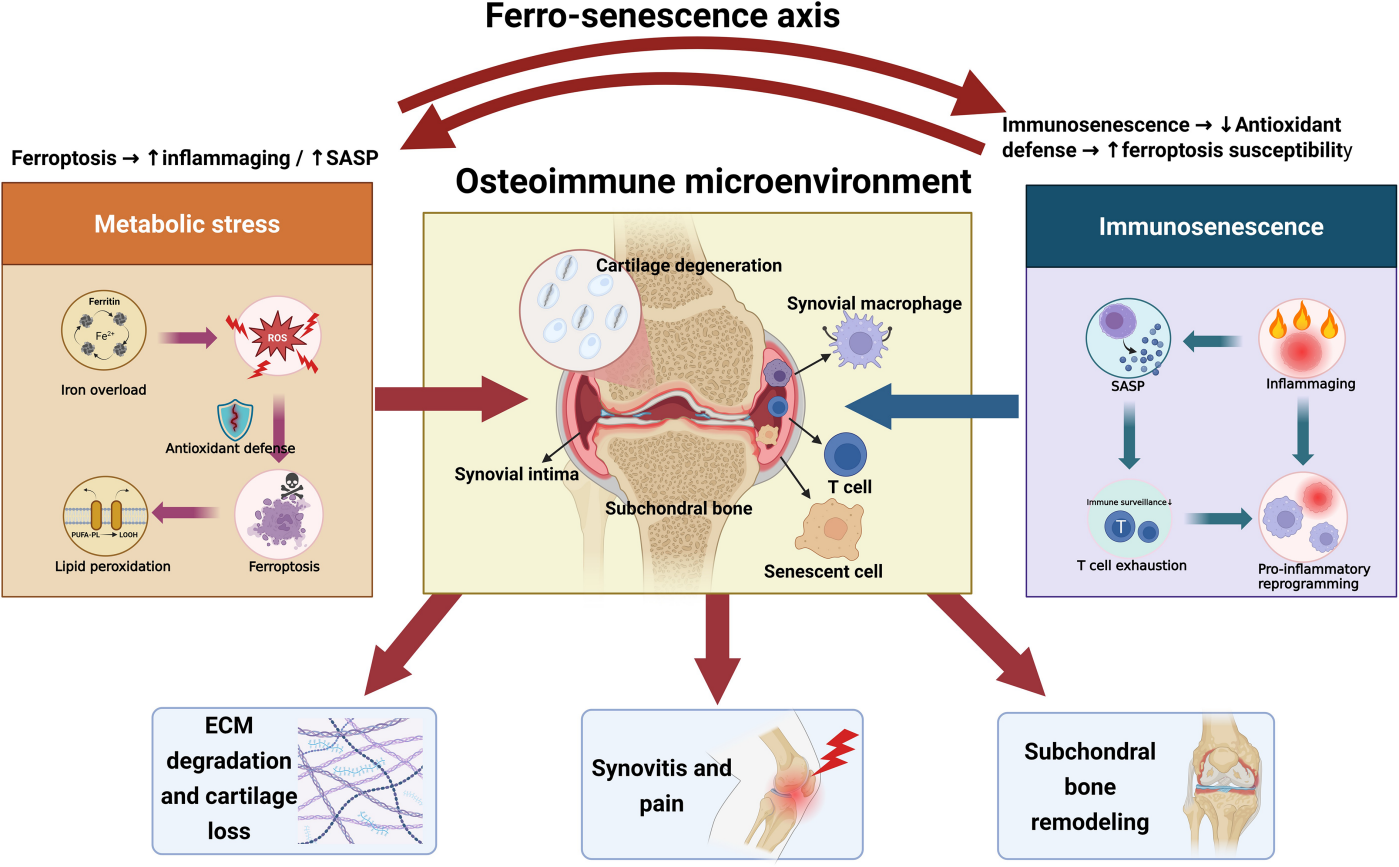
Proposed osteoimmune working model linking ferroptosis and immunosenescence in OA. This schematic summarizes the potential convergence of iron dysregulation, lipid peroxidation, impaired antioxidant defense, SASP-related inflammation, macrophage dysfunction, T-cell remodeling, chondrocyte injury, and subchondral bone changes within the OA osteoimmune microenvironment. The model integrates direct OA-related evidence with mechanistic inferences from other inflammatory, degenerative, and aging-related diseases and should therefore be interpreted as a hypothesis-generating framework requiring further OA-specific validation.

Based on this, the present review focuses on recent advances in the study of ferroptosis and immunosenescence in OA. It summarizes the roles of iron homeostasis imbalance, lipid peroxidation, antioxidant defense impairment, and immunosenescence-associated inflammatory remodeling in joint pathology, and further analyzes the potential crosstalk between these two processes in cartilage, synovium, subchondral bone, and related immune cells. At the same time, by considering both the strengths and limitations of current evidence, this review also outlines the current progress in relevant therapeutic strategies, with the aim of providing a reference for OA mechanistic research and future translational research. To avoid overinterpretation, the evidence discussed in this review is considered according to three levels. The first level includes mechanisms that have been directly demonstrated in OA tissues, OA animal models, or OA-related joint cells, such as iron overload, lipid peroxidation, impaired GPX4/SLC7A11-dependent antioxidant defense, senescence-associated inflammatory remodeling, and macrophage or chondrocyte dysfunction. The second level includes mechanisms inferred from other disease contexts, including cancer, cardiovascular diseases, neurodegenerative disorders, rheumatoid arthritis, and general aging models, where ferroptosis and immune aging-related pathways have been more directly investigated. The third level includes hypothetical pathways that are biologically plausible but remain to be experimentally validated in OA, particularly the existence of a stable bidirectional ferroptosis-immunosenescence amplification loop within the osteoimmune microenvironment. Therefore, the proposed ferroptosis-immunosenescence axis should be understood as a testable framework rather than a fully established pathogenic mechanism (**Figure 1**).

### 1.1 Literature search strategy and study selection

This narrative review was based on a comprehensive literature search of PubMed, Web of Science, Scopus, and Google Scholar. The literature search covered studies published from the introduction of the ferroptosis concept in 2012 to March 2026, with additional relevant older studies included when they provided foundational information on osteoarthritis, immunosenescence, inflammaging, or iron metabolism. The main search terms included “osteoarthritis,” “ferroptosis,” “immunosenescence,” “inflammaging,” “cellular senescence,” “senescence-associated secretory phenotype,” “SASP,” “iron metabolism,” “iron overload,” “ferritinophagy,” “NCOA4,” “lipid peroxidation,” “GPX4,” “SLC7A11,” “chondrocyte,” “synovial macrophage,” “T cell,” “fibroblast-like synoviocyte,” “subchondral bone,” “osteoimmune microenvironment,” and “osteoimmunology,” either alone or in combination.

Studies were selected according to their relevance to the following topics: ferroptosis-related mechanisms in OA or OA-relevant cells; immunosenescence, cellular senescence, and SASP-related inflammatory remodeling in OA; immune–stromal interactions involving chondrocytes, synovial macrophages, T cells, fibroblast-like synoviocytes, and subchondral bone cells; and therapeutic strategies targeting ferroptosis, senescence, inflammation, or local delivery in OA. Priority was given to original research articles using human OA tissues, OA animal models, or OA-related joint cells, as well as recent high-quality reviews that summarized key mechanistic advances. Studies from cancer, cardiovascular diseases, neurodegenerative disorders, rheumatoid arthritis, and general aging models were included when they provided mechanistic insights relevant to ferroptosis, immune aging, or their potential interaction, but these findings were interpreted as indirect evidence unless validated in OA-specific settings. Articles with limited mechanistic relevance, insufficient methodological detail, or only peripheral relevance to the ferroptosis–immunosenescence framework were not emphasized.

## 2 Dual crises in the osteoimmune microenvironment

### 2.1 The metabolic landscape of ferroptosis: collapse of iron, lipids, and defensive barriers

#### 2.1.1 Iron overload and the Fenton reaction

In patients with OA, iron overload often arises from intra-articular hemorrhage, synovial microvascular proliferation, or systemic iron metabolism disorders, leading to abnormal accumulation of the labile iron pool (LIP) in joint tissues and accelerating chondrocyte degeneration (12). In healthy joints, iron uptake, storage, and export are tightly regulated. In recent years, increasing evidence has shown that iron overload plays an important role in OA progression by aggravating joint injury through the induction of oxidative stress and cell death. Excess ferrous iron (Fe²^+^) can promote Fenton chemistry, thereby enhancing ROS generation, lipid peroxidation, and cellular damage (13). This process has been widely observed in the synovial fluid and tissues of patients with OA and is particularly associated with cartilage degradation, synovial inflammation, and subchondral bone remodeling (14). In guinea pig models, systemic iron overload significantly aggravated OA progression, as reflected by accelerated cartilage degradation and enhanced joint inflammation (15). A clinical cross-sectional study based on NHANES further confirmed a positive association between iron overload and OA incidence, suggesting that iron overload may increase the risk of OA by disrupting joint homeostasis through iron-induced oxidative damage, thereby identifying iron overload as a risk factor for osteoarthritis (16). In synovial tissue, the detrimental effects of iron overload appear to be particularly pronounced. Synovial microvascular proliferation may increase local iron deposition and expand the labile iron pool, thereby reinforcing oxidative stress (17). Studies have shown that iron overload induces oxidative stress, cell-cycle arrest, and apoptosis in chondrocytes and disrupts the cartilage matrix through ROS-mediated mechanisms (18). In addition, in knee OA models, intracellular iron overload may promote the progression of hemochromatosis-related OA by driving M1 polarization of synovial macrophages, and may aggravate ferroptosis and articular cartilage injury through activation of the mTORC1-p70S6K/4E-BP1 pathway (19). Iron intake has also been linked to OA progression, and excessive iron intake may worsen the disease through similar mechanisms. These alterations are not confined to local tissues, but may also involve systemic effects. At the cellular level, this iron imbalance favors lipid peroxidation and ferroptosis-related chondrocyte injury (20). Single-cell RNA sequencing studies have revealed the presence of iron overload–associated chondrocyte subpopulations with abnormal iron metabolism in hand OA, further supporting the contribution of iron overload to OA heterogeneity (21). Several experimental compounds have shown protective effects in OA-related models by modulating iron homeostasis and redox pathways. Biochanin A directly reduces intracellular iron levels by inhibiting transferrin receptor 1 (TfR1) and promoting ferroportin (FPN), while also targeting the Nrf2/System Xc^-^/GPX4 signaling pathway to eliminate free radicals and prevent lipid peroxidation, thereby protecting against knee OA progression by restoring iron homeostasis (22). Naringenin alleviates chondrocyte injury under iron overload conditions and attenuates OA progression by reducing oxidative stress through the NRF2-HO-1 pathway (23). Paeoniflorin significantly improves chondrocyte viability through the p53/SLC7A11/GPX4 pathway, restores matrix metabolism, reduces iron accumulation and oxidative stress, preserves mitochondrial function under iron overload conditions, and mitigates iron overload–induced OA progression (24). These findings suggest that iron overload is not only a risk factor for OA but also a potential therapeutic target, and that modulation of the Fenton reaction and the labile iron pool may improve disease progression. Overall, iron overload represents an important pro-oxidative factor in OA and provides a metabolic basis for ferroptosis-related injury.

#### 2.1.2 Lipid peroxidation and membrane fragility

As an iron-dependent form of regulated cell death, ferroptosis is characterized by lipid peroxidation and increased membrane fragility and plays a key role in OA progression. Lipid peroxidation mainly involves oxidative damage to PUFA-containing membrane phospholipids and is a central execution event in ferroptosis (25, 26). In OA, lipid peroxidation may disrupt chondrocyte membrane integrity and promote inflammatory signaling through DAMP release (27). Studies have shown that lipid peroxidation directly influences cell death outcomes, including the balance among apoptosis, autophagy, and ferroptosis, and that ROS-driven lipid peroxidation can shift ferroptosis-prone cells toward irreversible membrane damage (28). Aberrant activation of acyl-CoA synthetase long-chain family member 4 (ACSL4) and lysophosphatidylcholine acyltransferase 3 (LPCAT3) is a key mechanism underlying amplification of lipid peroxidation. These enzymes promote the esterification of polyunsaturated fatty acids (PUFAs) into membrane phospholipids, thereby rendering the membrane highly susceptible to peroxidation (29). As an essential mediator of ferroptosis, ACSL4 activates arachidonic acid (AA) and adrenic acid (AdA) and incorporates them into phosphatidylethanolamine (PE), generating PUFA-PLs (30). In OA cartilage, ACSL4 is markedly upregulated, resulting in altered membrane lipid composition enriched in oxidizable PUFAs and thereby placing cells in a ferroptosis-prone state (31). LPCAT3 acts in concert with ACSL4 to insert these PUFAs into membrane structures, further increasing membrane susceptibility to peroxidative damage. When ROS attack membrane-associated PUFAs, lipid hydroperoxides (L-OOH) are generated (32). If not promptly eliminated, L-OOH decomposes into cytotoxic aldehydes, such as 4-hydroxynonenal (4-HNE) and malondialdehyde (MDA). 4-HNE has been described as a “second toxic messenger,” as it not only disrupts membrane integrity but also forms adducts with proteins and DNA, leading to cellular dysfunction or even cell death (33). In cancer and cardiovascular disease models, overexpression of ACSL4 and LPCAT3 enhances ferroptosis sensitivity, whereas inhibition of their activity alleviates lipid peroxidation-induced changes in membrane fragility (34, 35), suggesting that similar mechanisms may also be relevant in OA.

#### 2.1.3 Collapse of antioxidant systems and defensive axes

Under physiological conditions, cells rely on glutathione peroxidase 4 (GPX4) to reduce toxic lipid hydroperoxides (L-OOH) into non-toxic lipid alcohols (L-OH), thereby blocking ferroptosis. However, multiple factors in the OA microenvironment contribute to the collapse of this defense system. System Xc^-^ supports cystine uptake and GSH synthesis; its dysfunction weakens GPX4-dependent lipid peroxide clearance (9). In OA, inhibition of System Xc^-^ is often driven by inflammatory mediators and oxidative stress, resulting in downregulation of solute carrier family 7 member 11 (SLC7A11), a core subunit of System Xc^-^. Thus, impairment of the System Xc^-^/GSH/GPX4 axis increases chondrocyte susceptibility to ferroptosis (36). In OA models, modulation of the Xc^-^/GPX4 axis has been shown to significantly reduce cell death and suppress ferroptosis progression (37). In addition, inhibition of System Xc^-^ is closely linked to nuclear factor erythroid 2-related factor 2 (NRF2) signaling. NRF2 is an important transcriptional regulator of anti-ferroptotic genes, including GPX4 and System Xc^-^, and plays a central role in preventing lipid peroxidation and free iron accumulation. As a stress-inducible transcription factor, NRF2 translocates into the nucleus and promotes transcription of antioxidant response element (ARE)-containing genes, many of which are responsible for limiting lipid peroxidation and ferroptosis (38). GSH depletion further compromises GPX4 activity and reinforces ferroptosis susceptibility (39). In OA, GSH depletion may result from both System Xc^-^ dysfunction and excessive ROS generation. Reduced GSH levels have been associated with OA severity and may exacerbate cartilage damage by weakening the Xc^-^/GSH/GPX4 axis (40). Activation of the NRF2/GPX4 axis has been reported to reduce oxidative stress, lipid peroxidation, and ferroptosis-related cartilage damage in OA models (41). Likewise, activation of the sirtuin 1 (SIRT1)/NRF2 signaling pathway has been reported to increase GSH production and upregulate GPX4 and SLC7A11 expression in interleukin-1β (IL-1β)-stimulated chondrocytes, significantly inhibiting inflammatory responses and ferroptosis and thus attenuating OA progression (42). Mechanical stress is another critical factor in OA pathogenesis. Moderate mechanical stress may activate the NRF2 antioxidant system and suppress nuclear factor kappa B (NF-κB) p65 signaling, thereby alleviating chondrocyte ferroptosis (43). However, in the OA microenvironment, abnormal or excessive mechanical stress, such as joint overloading, can lead to collapse of antioxidant defenses, increased ferroptosis sensitivity, and induction of chondrocyte ferroptosis, thereby accelerating OA progression (44). Mechanical stress can also regulate GPX4 expression. Experiments in human OA cartilage and murine OA chondrocytes have shown that persistent mechanical stress downregulates GPX4, promotes chondrocyte ferroptosis, and aggravates OA severity (45). In addition, mechanical stress may activate dynamin-related protein 1 (Drp1)-mediated mitochondrial fission, leading to mitochondrial fragmentation, accumulation of mitochondrial ROS, mitochondrial dysfunction, lipid peroxidation, and ferroptosis, thereby further exacerbating inflammation (46).

### 2.2 Immunosenescence in the osteoimmune microenvironment: from functional exhaustion to pro-inflammatory reprogramming

#### 2.2.1 Inflammaging: the pro-inflammatory background tone of the osteoimmune microenvironment

Inflammaging is one of the most representative phenotypes of immunosenescence and is characterized by a chronic, low-grade, sterile inflammatory state that persists in the absence of overt infection or autoimmune triggers. In OA joints, this inflammatory state is not a transient protective response, but rather the cumulative result of aging, sustained mechanical loading, and metabolic stress (6, 47). In OA and other age-related diseases, cells can enter a senescence program. Multiple cell types, including senescent immune cells, fibroblast-like synoviocytes (FLS), and damaged chondrocytes, gradually acquire a senescence-associated secretory phenotype (SASP) (48, 49). Representative SASP factors, such as L-1β, interleukin-6 (IL-6), and tumor necrosis factor-α (TNF-α), constitute a persistent pro-inflammatory “background signal” in OA synovial fluid (50). Through inflammatory cytokines, chemokines, and proteases, SASP factors contribute to cartilage catabolism, synovial inflammation, and pain sensitization (51). They can promote chondrocyte catabolic responses, including upregulation of MMPs and ADAMTS family members, thereby accelerating extracellular matrix degradation, impairing the function of bone-related cells, disrupting skeletal homeostasis, and promoting skeletal aging. At the same time, persistent activation of NF-κB and mitogen-activated protein kinase (MAPK) signaling, together with oxidative stress-induced mitochondrial dysfunction and epigenetic modifications, prolongs the release of SASP factors, thereby reinforcing inflammation, structural damage, and senescence propagation in cartilage and synovium (52, 53). These mediators may also weaken anti-ferroptotic defenses, including the System Xc^-^–GSH–GPX4 axis, thereby potentially lowering the threshold for ferroptosis, thereby potentially lowering the threshold for ferroptosis, although direct OA-specific evidence linking SASP-driven inflammatory remodeling to ferroptosis susceptibility still requires further validation (54). More importantly, inflammaging exhibits a marked self-amplifying nature. SASP not only acts on neighboring cells, but can also reinforce senescence programs within immune cells themselves, thereby forming a positive feedback loop of “inflammation–senescence–re-inflammation.” Within the joint cavity, this process may provide an immunological background for chronic OA progression (55).

#### 2.2.2 Senescence-associated reprogramming of synovial macrophages: coupling of polarization imbalance and iron metabolism

Synovial macrophages are key immune cells involved in joint surveillance, homeostasis, and inflammatory remodeling (56). In OA synovium, senescent macrophages exhibit enhanced M1 polarization, mitochondrial injury, and impaired efferocytosis, which may increase the SASP within the joint and thereby further aggravate OA (57). Targeted inhibition of the release of pro-inflammatory and catabolic factors derived from senescent macrophages has been shown to shift chondrocytes from a catabolic state toward an anabolic state, thereby slowing OA progression (58). In OA, senescence-associated macrophage remodeling may be tightly coupled with local iron dysregulation. With aging or recurrent microbleeding, synovial macrophages may accumulate iron through phagocytosis of blood-derived products and impaired iron export. Iron overload may act as a pro-inflammatory signal that favors M1-like macrophage polarization. Studies have shown that intracellular iron accumulation can strongly promote the shift of macrophages toward a pro-inflammatory M1 phenotype by activating the mTORC1–ribosomal protein S6 kinase beta-1 (p70S6K)/eukaryotic translation initiation factor 4E-binding protein 1 (4E-BP1) signaling pathway and enhancing p53 acetylation (19, 59). Senescent and iron-loaded M1-like macrophages may serve as a source of SASP-related inflammatory mediators and hepcidin. Among these mediators, hepcidin induces degradation of FPN on the surface of chondrocytes, thereby aggravating intracellular iron retention in chondrocytes and suggesting a potential feed-forward interaction between iron metabolism and inflammatory signaling along the synovium–cartilage axis (60). In addition to sustained release of pro-inflammatory mediators, immunosenescence also markedly weakens macrophage efferocytosis, namely, the clearance of apoptotic cells or ferroptotic cell debris (61). Uncleared cellular remnants may release DAMPs and promote pro-inflammatory macrophage activation, including NLRP3-related signaling, thereby further enhancing inflammatory responses (62). For example, high-mobility group box 1 (HMGB1) released from M2 macrophages can interact with toll-like receptor 4 (TLR4) on M1 macrophages, thereby triggering signal transducer and activator of transcription 3 (STAT3) signaling in M1 macrophages and promoting inflammatory responses. This mechanism, mainly described in iron-rich arthritic contexts, may provide a model for future validation in OA synovium (63).

#### 2.2.3 Degeneration of adaptive immunity

**Figure 2:**
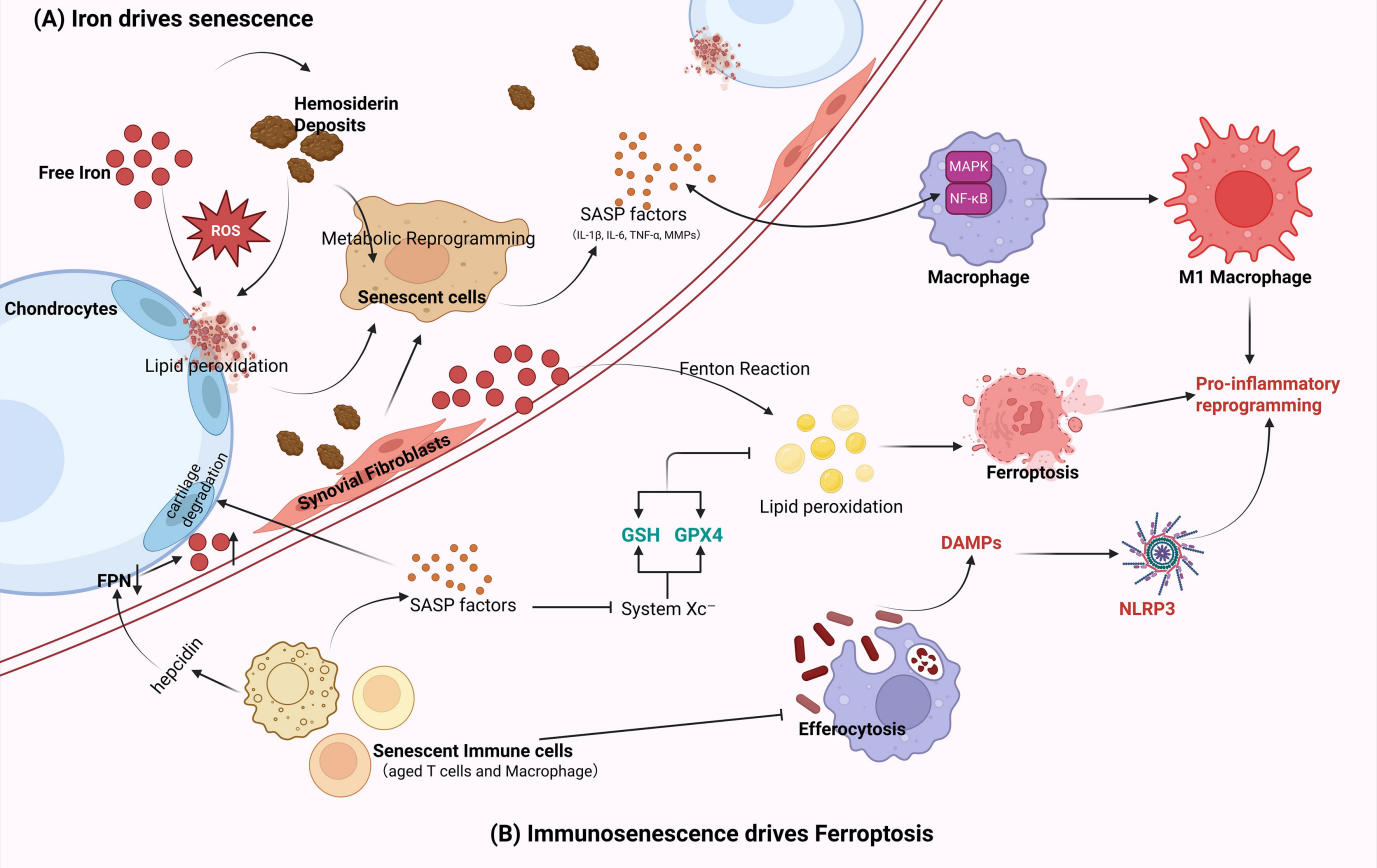
Potential bidirectional interplay between iron-driven senescence and immunosenescence-associated ferroptosis in the osteoimmune microenvironment of osteoarthritis.

Aging is accompanied by profound changes in the immune system, particularly within the T-cell compartment (64). The aged adaptive immune system is characterized by progressively worsening dysfunction and increased autoimmune tendency. With advancing age, intrinsic alterations in CD4^+^ T cells can drive chronic inflammation and are sufficient to accelerate the emergence of systemic aging phenotypes (65). During immunosenescence, T cells gradually shift from naïve or memory phenotypes with high proliferative potential and immune surveillance capacity toward terminally differentiated senescent phenotypes characterized by loss of the costimulatory molecule CD28 and increased expression of CD57 (CD28^-^CD57^+^) (66). In both peripheral blood and synovial fluid from patients with OA, the proportions of CD4^+^CD28^-^ and CD8^+^CD28^-^ T-cell subsets are significantly increased (67). These cells show little effective proliferation in response to antigen stimulation, and their T-cell receptor (TCR) signaling is impaired, indicating a decline in immune surveillance function (68). At the same time, this loss of function is accompanied by markedly enhanced pro-inflammatory potential. These senescent T cells undergo clonal expansion in synovial tissue and secrete large amounts of interferon-γ (IFN-γ), interleukin-17 (IL-17), and TNF-α, while also releasing perforin and granzymes, thereby causing bystander damage to local joint tissues and exacerbating chronic inflammation and joint destruction (69, 70). From a metabolic perspective, T-cell senescence is closely associated with lipid metabolic reprogramming and oxidative stress. T-cell survival and effector function also depend on lipid peroxide control, including GPX4-mediated antioxidant defense. Once GPX4 activity is impaired, endogenous ROS generated during activation rapidly trigger accumulation of membrane lipid peroxides, leading to ferroptosis or functional exhaustion of peripheral T cells (71). Studies in the tumor microenvironment have shown that increased lipid peroxidation in CD8^+^ T cells promotes T-cell exhaustion and tumor progression by activating the p38 kinase pathway and reducing transcription of effector cytokine genes, whereas GPX4 overexpression in CD8^+^ T cells reduces lipid peroxidation, rescues T-cell function, and enhances antitumor activity (72). Ferroptosis driven by lipid peroxidation is therefore considered an important mechanism underlying CD8^+^ T-cell dysfunction. Although direct OA evidence remains limited, these findings suggest a possible link between oxidized lipid stress and T-cell dysfunction that warrants OA-specific validation. A bioinformatic analysis of senescence-related genes in OA further found that some senescence-associated genes were closely correlated with infiltration of neutrophils, plasmacytoid dendritic cells, activated CD4^+^ T cells, and type 2 helper T cells, with particularly marked changes during OA progression, suggesting the potential value of senescence-targeted personalized strategies for senescence-associated OA (73). It is also worth noting that different T-cell subsets do not exhibit the same sensitivity to ferroptosis. Recent studies have shown that interleukin-9 (IL-9)-secreting CD8^+^ Tc9 cells display lower levels of lipid peroxidation and greater resistance to ferroptosis than classical Tc1 cells, a feature associated with enhanced mitochondrial fatty acid oxidation (FAO) mediated by IL-9/STAT3 signaling (74). These findings provide a rationale to investigate whether metabolic reprogramming can preserve adaptive immune function in OA joints. By modulating the lipid metabolic state of T cells, it may be possible to partially recover their function in OA under conditions of immunosenescence. Overall, immunosenescence in OA reflects a profoundly imbalanced state: immune clearance and surveillance progressively decline, whereas pro-inflammatory and pro-degenerative signaling continues to intensify. It is this immunosenescence-driven reprogramming of the osteoimmune microenvironment that may create favorable conditions for the initiation and amplification of ferroptosis, thereby providing a possible immunological context in which iron dysregulation and senescence-related remodeling may converge (**Figure 2**).

## 3 Possible crosstalk between ferroptosis and immunosenescence in osteoarthritis: mechanistic links, evidence hierarchy, and unresolved questions

Before discussing the possible mechanistic links between ferroptosis and immunosenescence in OA, it is necessary to clarify the strength and boundaries of the available evidence. At present, direct OA-specific evidence is strongest for iron dysregulation, lipid peroxidation, oxidative stress, inflammatory remodeling, chondrocyte injury, and macrophage dysfunction. In contrast, several proposed connections between ferroptosis and immune aging are still mainly inferred from studies in cancer, rheumatoid arthritis, cardiovascular diseases, neurodegenerative disorders, and general aging models. To improve interpretability, the proposed mechanisms are stratified according to the main source of supporting evidence, including human OA data, animal OA models, OA-related *in vitro* studies, evidence inferred from non-OA disease contexts, and hypothesis-generating mechanisms that remain to be experimentally validated in OA. Therefore, the following discussion should be interpreted according to an evidence hierarchy rather than as proof of a fully established bidirectional causal loop. To make this distinction explicit, the major proposed mechanisms are summarized in **Table 1**.

**Table 1:** Evidence hierarchy of proposed ferroptosis–immunosenescence interactions in osteoarthritis.

Proposed interaction	OA-specific evidence	Main inferred evidence from other contexts	Evidence status	Key validation need in OA
Iron overload promotes oxidative stress, lipid peroxidation, and joint cell injury	Iron accumulation, altered ferritin levels, oxidative stress, lipid peroxidation, and cartilage degeneration have been reported in OA tissues, animal models, and OA-related cells.	Iron-driven ferroptosis and oxidative injury are well established in cardiovascular, neurodegenerative, fibrotic, and aging-related diseases.	Human OA data + animal OA models	Identify the OA cell populations and disease stages most sensitive to iron overload.
Iron accumulation promotes pro-inflammatory macrophage remodeling	Iron overload has been linked to M1-like polarization of synovial macrophages and aggravated cartilage injury in OA models.	Iron-dependent macrophage inflammatory reprogramming has also been observed in metabolic and inflammatory diseases.	Human OA data + animal OA models	Determine whether iron retention directly induces senescence-like macrophage phenotypes in OA synovium.
Senescence-associated inflammation lowers anti-ferroptotic capacity	OA joints show persistent SASP-related cytokines, oxidative stress, and impaired redox homeostasis.	IFN-γ, TNF-α, IL-1β, and IL-6 can suppress the SLC7A11/GSH/GPX4 axis and increase ferroptosis susceptibility in cancer and inflammatory disease models.	Human/animal OA evidence + *in vitro* support	Test whether senescent immune or stromal cells directly reduce anti-ferroptotic defenses in OA chondrocytes, FLS, or bone-lineage cells.
Impaired efferocytosis amplifies DAMP-driven inflammation	Dysfunctional OA synovial macrophages show impaired phagocytic/efferocytic capacity and enhanced inflammatory activity.	Ferroptotic cell debris and DAMPs can activate TLR4, RAGE, NF-κB, and NLRP3-related inflammatory pathways in other disease models.	Human/animal OA evidence + *in vitro* support	Determine whether defective efferocytosis causes accumulation of ferroptotic cells or ferroptosis-derived DAMPs in OA joints.
Ferroptosis-related macrophage imbalance contributes to persistent inflammation	Synovial macrophage polarization is closely associated with OA synovitis, cartilage catabolism, and disease progression.	Selective ferroptosis of M2-like macrophages and HMGB1-mediated activation of M1 macrophages have been suggested mainly in arthritis or non-OA inflammatory models.	Mainly inferred from non-OA diseases	Verify whether selective M2-like macrophage ferroptosis occurs in OA synovium and sustains M1 dominance.
Ferroptotic chondrocytes release immune-modulatory signals	Lipid peroxidation products such as 4-HNE and MDA are increased in OA and are associated with cartilage degeneration and inflammatory signaling.	Ferroptosis-derived mediators, including lipid peroxidation products and HMGB1, can regulate NF-κB, MAPK, TLR4, STING, and inflammasome pathways in other diseases.	Human/animal OA evidence + *in vitro* support	Define which ferroptosis-derived signals from OA chondrocytes directly activate synovial macrophages, FLS, or T cells.
T-cell immunosenescence is linked to ferroptosis-related metabolic stress	Senescence-like T-cell subsets and altered adaptive immune responses have been reported in OA blood, synovial fluid, or synovial tissue.	GPX4 deficiency, oxidized lipid uptake, CD36-p38 signaling, and lipid peroxidation can impair T-cell survival and effector function in cancer, aging, and immune disease models.	Mainly inferred from non-OA diseases	Determine whether OA-infiltrating T cells undergo ferroptosis or ferroptosis-related functional exhaustion.
NCOA4-mediated ferritinophagy connects iron dysregulation, ferroptosis, and senescence	Iron dysregulation and ferritin alteration are relevant to OA, but direct evidence linking NCOA4-mediated ferritinophagy to immunosenescence in OA remains limited.	Ferritinophagy contributes to labile iron release, ferroptosis, and senescence-related injury in aging, fibrosis, and organ degeneration models.	Mainly inferred from non-OA diseases	Test whether NCOA4-dependent ferritinophagy drives SASP acquisition or senescence-related remodeling in OA cells.
Subchondral bone ferroptosis contributes to inflammatory remodeling	Ferroptosis-related changes, endothelial dysfunction, vascular leakage, abnormal angiogenesis, and subchondral bone remodeling have been implicated in OA progression.	Endothelial ferroptosis, vascular permeability, immune cell entry, and inflammatory remodeling are better defined in vascular and degenerative disease models.	Human/animal OA evidence + *in vitro* support	Clarify whether ferroptosis in subchondral bone endothelial or bone-lineage cells directly promotes immune aging-like remodeling in OA.
A bidirectional ferroptosis–immunosenescence amplification loop exists in OA	Ferroptosis-related injury and immunosenescence-related inflammatory remodeling coexist in OA and may converge through iron dysregulation, oxidative stress, lipid peroxidation, and SASP-related signaling.	Reciprocal interactions among ferroptosis, inflammation, immune dysfunction, and senescence are supported by studies in cancer, rheumatoid arthritis, cardiovascular disease, neurodegeneration, and aging models.	Hypothesis-generating; no direct OA validation	Establish temporal, spatial, and cell type-specific causal evidence for a bidirectional interaction within OA tissues.

OA, osteoarthritis; SASP, senescence-associated secretory phenotype; FLS, fibroblast-like synoviocytes; GSH, glutathione; GPX4, glutathione peroxidase 4; SLC7A11, solute carrier family 7 member 11; DAMPs, damage-associated molecular patterns; TLR4, Toll-like receptor 4; RAGE, receptor for advanced glycation end products; NF-κB, nuclear factor-kappa B; NLRP3, NOD-like receptor family pyrin domain-containing 3; HMGB1, high-mobility group box 1; 4-HNE, 4-hydroxynonenal; MDA, malondialdehyde; MAPK, mitogen-activated protein kinase; STING, stimulator of interferon genes; NCOA4, nuclear receptor coactivator 4.

Based on this evidence hierarchy, the following sections discuss three major potential routes of interaction: iron overload as a metabolic driver of senescence-related changes, immunosenescence-associated inflammatory remodeling as a potential factor lowering the threshold for ferroptosis, and ferroptosis-related products as possible mediators of immune activation and inflammatory amplification. Particular attention is given to whether each mechanism has been directly demonstrated in OA or remains to be validated in OA-specific models. It should be emphasized that the coexistence of iron overload, oxidative stress, ferroptosis-related injury, and immunosenescence-associated remodeling in OA does not necessarily indicate direct mechanistic coupling. These processes may occur in parallel as convergent responses to aging, chronic mechanical stress, metabolic imbalance, and low-grade inflammation. Therefore, the term “crosstalk” in this review is used to describe a biologically plausible interaction framework rather than a fully proven bidirectional causal pathway. Where available, priority is given to studies providing intervention-based evidence, such as genetic manipulation, pharmacological inhibition, rescue experiments, or OA animal model validation. In contrast, mechanisms supported only by clinical association, transcriptomic correlation, *in vitro* observation, or evidence from non-OA disease contexts are interpreted as associative or hypothesis-generating and require further OA-specific validation.

### 3.1 Iron overload: an important metabolic driver of cellular senescence

Beyond its role in ferroptosis-related injury, iron overload may also act as a chronic metabolic stressor that contributes to senescence-related changes. In OA, dysregulated iron homeostasis is considered to be closely associated with senescence of chondrocytes and synovial cells, giving rise to a senescent phenotype characterized by iron-dependent oxidative damage and functional decline (75). Clinical studies have shown that higher ferritin levels in elderly populations are associated with an increased risk of OA, and that ferritin levels are positively correlated with the severity of joint damage (13, 76). These findings suggest that iron accumulation may not merely be an accompanying phenomenon, but may also participate in OA pathogenesis. As noted above, excess Fe²^+^ can sustain oxidative stress through Fenton chemistry. This persistent redox stress may damage macromolecules and mitochondria, thereby activating DNA damage and senescence-associated pathways (77). Persistent oxidative stress may further cause DNA double-strand breaks and mitochondrial DNA damage, activate classical DNA damage response pathways such as ataxia telangiectasia mutated/ATM and Rad3-related (ATM/ATR)-p53-p21, and ultimately lead to permanent cell-cycle arrest and the establishment of a senescent phenotype (78–81). These changes may shift injured cells toward a senescence-like phenotype characterized by p53/p21 activation and SASP acquisition. Within the OA joint microenvironment, iron overload-driven senescent changes are not restricted to chondrocytes, but may also affect FLS. Studies have shown that synovial tissues with increased iron burden can release pro-inflammatory mediators such as IL-1β and TNF-α, thereby stimulating the catabolic activity of chondrocytes and accelerating cartilage degeneration (75). At the same time, iron overload may induce an SASP-like phenotype in FLS through ROS-dependent mechanisms, upregulating molecules such as IL-6 and matrix metalloproteinase-13 (MMP-13), and thereby promoting joint inflammation and fibrosis (13, 82). In addition, Fe²^+^ may regulate the stability of hypoxia-inducible factor-1α (HIF-1α) by affecting prolyl hydroxylase domain protein 2 (PHD2) activity and promoting vascular endothelial growth factor (VEGF) secretion, thereby contributing to synovial pannus formation and pathological vascular remodeling (83). Together, these findings suggest that iron overload may connect oxidative stress with synovium–cartilage inflammatory communication. NCOA4-mediated ferritinophagy may provide another mechanism linking iron release to ferroptosis- and senescence-related stress. Under physiological conditions, NCOA4 recognizes and transports ferritin to lysosomes for degradation, thereby finely regulating intracellular iron release. When this process becomes abnormal, disturbed iron homeostasis may aggravate labile iron accumulation and further amplify oxidative damage and the senescent phenotype. Existing studies have shown that interference with NCOA4-mediated ferritinophagy can, to some extent, alleviate iron overload-induced cellular senescence (84–86).It should be noted that the link between iron accumulation and cellular senescence has been relatively well supported in fibrotic diseases and age-related organ injury, whereas direct evidence in OA is still mainly confined to cartilage- and synovium-related cells. In other words, although iron overload-driven senescence in OA has strong biological plausibility, the specific cell types involved, their relative contributions at different disease stages, and whether this process is sufficient to drive immunosenescence remodeling at the whole-joint level all require further clarification through *in vivo* and cell type-specific studies.

### 3.2 Immunosenescence may reduce the anti-ferroptotic capacity of joint cells through inflammatory remodeling

Immunosenescence is not merely characterized by weakened immune responses; more importantly, it represents a pathological state marked by persistent chronic low-grade inflammation, immune cell dysfunction, and abnormally enhanced paracrine signaling. In this context, senescent macrophages and T cells can continuously release a variety of SASP factors, such as IFN-γ, TNF-α, IL-1β, and IL-6, thereby altering the redox environment and metabolic state of local joint cells. Current evidence suggests that this persistent pro-inflammatory stimulation may weaken the antioxidant defenses of surrounding cells, increase the burden of lipid peroxidation, and lower their threshold for tolerating ferroptosis. Mechanistically, senescence-associated inflammatory mediators may promote susceptibility to ferroptosis through multiple pathways. First, IFN-γ and TNF-α can inhibit the solute carrier family 7 member 11 (SLC7A11)/GSH/GPX4 antioxidant axis, thereby restricting cystine uptake and impairing GSH synthesis, which in turn reduces the capacity to clear lipid peroxides (87–89). On this basis, ROS progressively accumulate and may exceed the buffering capacity of the cellular antioxidant system, ultimately driving membrane phospholipid peroxidation and ferroptosis (90). Therefore, the chronic pro-inflammatory environment sustained by immunosenescence may amplify joint cell injury by suppressing anti-ferroptotic defenses. In addition, nitric oxide (NO) and its derived reactive nitrogen species may also participate in this process within the inflammatory joint microenvironment. NO can react with superoxide anions to generate peroxynitrite (ONOO^-^), which has strong oxidative capacity and can induce lipid peroxidation, protein nitration, and oxidative DNA damage (90, 91). At the same time, NO may affect the activity of iron regulatory protein 1/iron regulatory protein 2 (IRP1/IRP2), thereby altering the expression of transferrin receptor and ferritin and disrupting intracellular iron metabolism (92). However, it should be noted that the role of NO in ferroptosis is highly context-dependent; in some cell models, it may also inhibit ferroptosis by terminating lipid peroxidation chain reactions (93). Therefore, NO is better regarded as a factor that may modulate ferroptosis sensitivity rather than as a simple unidirectional pro-ferroptotic molecule. At the macrophage level, increased TNF-α expression under senescent conditions can further activate the p38 MAPK pathway, increase intracellular ROS accumulation, promote pro-inflammatory polarization and ferroptosis-related injury, and indirectly impair the repair capacity of surrounding bone- and cartilage-related cells (94, 95). In addition, some studies have found that exosomal components derived from macrophage foam cells can promote inflammation and ferroptosis through regulation of the PI3K/AKT/mTOR signaling pathway (96). These findings suggest that immune cells may influence the ferroptosis sensitivity of surrounding tissues not only through soluble inflammatory mediators, but also through extracellular vesicle-mediated mechanisms. In OA animal models, as the disease progresses, oxidative stress levels, senescence markers such as p53, p21, and p16, and inflammatory cytokines such as IL-1β, TNF-α, and IL-6 are all significantly increased in cartilage tissue (96, 97). This suggests that the immunosenescence-associated inflammatory environment and chondrocyte senescence and injury tend to worsen in parallel. More notably, previous studies have shown that macrophages in the quadriceps of OA mice display a pronounced senescent phenotype and can induce ferroptosis and muscle atrophy in skeletal muscle cells by suppressing the mTORC1-HMGCR pathway and reducing coenzyme Q10 synthesis (82). This finding suggests that immunosenescence may not only promote ferroptosis within the local joint microenvironment, but may also affect adjacent or even distant tissues through metabolic pathways.

### 3.3 Ferroptosis-related products may participate in immune regulation and amplify local inflammatory imbalance

**Figure 3:**
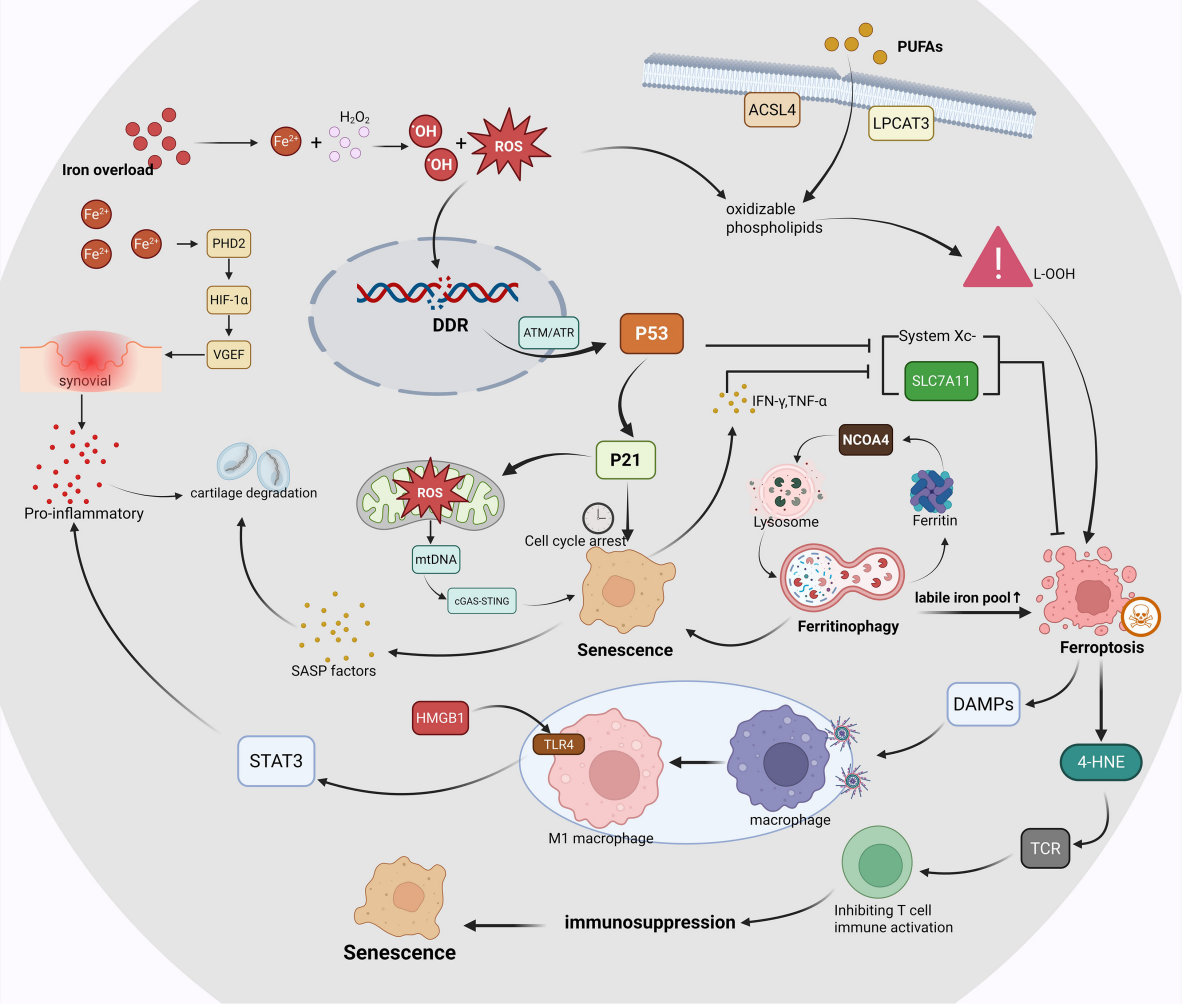
Possible crosstalk between ferroptosis and immunosenescence in osteoarthritis: Mechanistic Links and Evidence Boundaries.

Ferroptosis is not only a mode of cell death; its associated products may themselves act as immunoregulatory signals involved in the modulation of inflammation and immune responses. Within the disease microenvironment, multiple molecules released during ferroptosis can activate immune cells through pattern recognition receptors (PRRs) and thereby influence immune reactions. During cell death, large amounts of HMGB1 are released extracellularly. As a typical DAMP, HMGB1 can activate NF-κB signaling in macrophages and other innate immune cells through receptors such as TLR4 and receptor for advanced glycation end products (RAGE), thereby promoting the production of pro-inflammatory cytokines and amplifying local inflammatory responses (98). In iron-rich arthritic environments, M2 macrophages appear to be more susceptible to ferroptosis than M1 macrophages, resulting in an imbalance in the M1/M2 ratio. Ferroptosis of M2 macrophages has been reported to influence arthritis progression through HMGB1-related signaling, mainly via interaction between released HMGB1 and TLR4 on M1 macrophages, which subsequently triggers STAT3 signaling in M1 macrophages and promotes inflammatory responses (63). In cerebral ischemia models, HMGB1 has also been shown to regulate not only immune cell activity but also hepcidin expression and iron homeostasis, thereby forming a positive feedback loop between immunity and iron metabolism (99). During OA progression, lipid oxidation products released from ferroptotic chondrocytes, such as 4-hydroxynonenal (4-HNE), are markedly increased (100). These molecules are not only markers of ferroptosis, but also regulators of inflammation through interaction with multiple signaling pathways, including NF-κB, NRF2, MAPK,TLR4, and STING, thereby affecting immune responses, cytokine production, and inflammasome activation. The immunological effects of 4-HNE may vary depending on its concentration and the cell type involved, making it a highly dynamic modulator of immune responses and inflammation (101). Lipid peroxidation products such as 4-HNE that accumulate in inflammatory microenvironments can not only induce oxidative damage in neighboring cells, but can also form adducts with surface and intracellular signaling proteins in T cells, interfere with TCR signaling, and suppress normal T-cell activation and proliferation, thereby exerting immunosuppressive effects in chronic inflammation and immune exhaustion (102). DAMPs released during ferroptosis can also be recognized by surrounding macrophages and promote their pro-inflammatory activation and M1-like polarization through pattern recognition systems such as TLRs and the NLRP3 inflammasome. This recognition not only promotes macrophage recruitment and activation, but also upregulates pro-inflammatory cytokine expression, drives M1 polarization, and forms an inflammatory feedback loop, thereby further amplifying local inflammatory conditions and iron metabolism imbalance. In turn, inflammatory mediators and oxidative stress-related molecules secreted by M1 macrophages may further lower the tolerance threshold of neighboring cells to ferroptosis, thus potentially forming a “ferroptosis–inflammation–ferroptosis” amplification loop within the inflammatory microenvironment (93, 103) (**Figure 3**).

### 3.4 An integrated osteoimmune model linking ferroptosis and immunosenescence in OA

Based on the evidence discussed above, ferroptosis and immunosenescence in OA can be integrated into a three-layer osteoimmune working model. The first layer is an injury-initiation layer driven by iron dysregulation, oxidative stress, lipid peroxidation, and impaired antioxidant defense. In OA joints, iron accumulation, mitochondrial dysfunction, excessive ROS production, and disruption of the SLC7A11/GSH/GPX4 axis may increase the susceptibility of chondrocytes, synovial cells, macrophages, and subchondral bone-related cells to ferroptosis-related injury. These events provide a metabolic and redox background in which tissue-resident cells become more vulnerable to inflammatory and mechanical stress. The second layer is an immune-amplification layer shaped by immunosenescence-associated remodeling. Senescent immune and stromal cells can produce SASP-related mediators, including IL-1β, IL-6, TNF-α, chemokines, and matrix-degrading enzymes, thereby sustaining low-grade inflammation in the joint microenvironment. This inflammatory background may further weaken anti-ferroptotic defenses, enhance oxidative stress, and reduce the capacity of synovial macrophages to clear damaged or dying cells. At the same time, ferroptosis-related products, including lipid peroxidation products and DAMPs, may activate innate immune pathways such as TLR4/RAGE/NF-κB and NLRP3-related signaling, thereby reinforcing macrophage activation and inflammatory amplification. The third layer is a tissue-remodeling and feedback layer involving cartilage, synovium, and subchondral bone. Ferroptosis-related chondrocyte injury may promote matrix degradation and release immune-active mediators, while senescent or metabolically reprogrammed synovial macrophages and fibroblast-like synoviocytes may further amplify cartilage catabolism and synovitis. In subchondral bone, ferroptosis-related endothelial dysfunction, vascular leakage, abnormal angiogenesis, and bone remodeling may facilitate immune cell entry and chronic inflammatory remodeling. Together, these processes may generate a self-reinforcing pathological environment in which redox imbalance, immune aging, ferroptosis-related injury, and tissue remodeling converge to promote OA progression. Importantly, this model should be interpreted as a conceptual and hypothesis-generating framework rather than as a fully proven bidirectional causal loop. Direct OA evidence is strongest for iron dysregulation, lipid peroxidation, inflammatory remodeling, chondrocyte injury, macrophage dysfunction, and subchondral bone changes. In contrast, several specific links, such as ferroptosis-related T-cell dysfunction, selective ferroptosis of macrophage subsets, NCOA4-mediated ferritinophagy-driven senescence, and ferroptosis-induced immune aging, remain largely inferred from other disease contexts. Future studies combining spatial transcriptomics, single-cell multi-omics, lineage tracing, conditional genetic models, and functional perturbation experiments are needed to determine whether ferroptosis and immunosenescence form a true bidirectional amplification loop in OA tissues.

## 4 Cellular hubs: key “nodes” in the microenvironment

### 4.1 Chondrocytes

Chondrocytes are the principal effector cells responsible for maintaining articular cartilage homeostasis and represent one of the most important pathological hubs in the osteoimmune microenvironment of OA. Their significance lies not only in their high sensitivity to iron overload, oxidative stress, and senescence, but also in the fact that, once injured, they can further influence synovium, subchondral bone, and surrounding immune cells through the release of inflammatory and matrix-degrading signals. Therefore, chondrocytes are not only important target cells jointly affected by ferroptosis and immunosenescence, but also key nodes in the amplification of local pathological signaling. Notably, senescent chondrocytes in OA display a seemingly paradoxical yet pathologically important feature. On the one hand, these cells are often accompanied by ferritin accumulation, increased ROS, and enhanced lipid peroxidation, indicating marked activation of ferroptosis-related metabolism; on the other hand, under certain conditions they show relative tolerance to ferroptosis induction (104). This phenomenon suggests that senescent chondrocytes are not merely passive victims of ferroptosis, but may acquire a certain adaptive survival advantage under persistent oxidative stress. Previous studies have shown that p21 is significantly upregulated in cartilage from patients with OA and in destabilization of the medial meniscus (DMM) mouse models, and that it can enhance the anti-ferroptotic capacity of senescent chondrocytes by regulating the stability of GPX4. When p21 is knocked down, senescent chondrocytes become markedly more sensitive to ferroptosis inducers such as erastin, accompanied by accelerated accumulation of lipid peroxides (105). On the other hand, downregulation of p21 expression can reduce ROS accumulation, inhibit activation of NF-κB signaling, and attenuate IL-1β-induced catabolic and inflammatory responses in chondrocytes, thereby improving OA phenotypes (106). These findings suggest that p21-related pathways may represent an important regulatory node linking cellular senescence, anti-ferroptotic defense, and cartilage degeneration. In addition to p21, senescent chondrocytes may maintain survival through other compensatory mechanisms. For example, overexpression of the glutamate transporter excitatory amino acid transporter 1 (EAAT1) in senescent chondrocytes can enhance resistance to ferroptosis by influencing glutamate metabolism and glutathione synthesis; inhibition of EAAT1 selectively induces ferroptosis in senescent chondrocytes and improves cartilage homeostasis in OA mouse models (104). In obesity-associated OA, the DNA damage-activated p53–forkhead box O3 (FOXO3) gene circuit also exhibits dual regulation of senescence and ferroptosis: p53 not only mediates senescence by promoting cell-cycle arrest, but also increases ferroptosis sensitivity by suppressing solute carrier family 7 member 11 (SLC7A11), whereas intra-articular injection of a lentivirus overexpressing FOXO3 significantly alleviates OA progression in obese mice (107).

Recent single-cell transcriptomic studies further suggest functional heterogeneity related to ferroptosis within the chondrocyte population. In knee OA cartilage, researchers identified a key ferroptosis-active subpopulation termed ferroptosis-active homeostasis chondrocytes (HomC). This subpopulation exhibits prominent lipid peroxidation features and a large number of ferroptosis-related differentially expressed genes, and has been recognized as a hub cell population that communicates intensively with FLS through fibroblast growth factor 1–fibroblast growth factor receptor 1 (FGF1–FGFR1) signaling (108). This signaling axis may not only promote activation of synovial inflammatory pathways, but may also be associated with extracellular matrix (ECM) remodeling and fibrosis. In addition to HomC, subpopulations such as InflamC and FC in hand OA also show enrichment of ferroptosis pathways and upregulation of iron overload-related genes (21). These findings indicate that chondrocytes in OA are not only recipients of oxidative damage and ferroptosis, but may also actively participate in inflammatory amplification and tissue remodeling through communication with the synovial and immune microenvironment. From the perspective of tissue injury, chondrocytes are highly sensitive to disturbances in iron homeostasis. Consistent with the mechanisms discussed above, iron dysregulation and impaired antioxidant defense may promote chondrocyte lipid peroxidation and matrix catabolism, as well as upregulate key catabolic mediators such as a disintegrin and metalloproteinase with thrombospondin motifs 5 (ADAMTS5) and MMP-13, thereby driving cartilage destruction. Application of the ferroptosis inhibitor ferrostatin-1 (Fer-1) can reduce oxidative stress in chondrocytes and decrease the expression of ADAMTS5 and MMP-13, suggesting that inhibition of ferroptosis may delay OA progression to some extent (9, 109).

In addition, mechanical stress, one of the core risk factors for OA initiation and progression, can convert abnormal loading into pro-ferroptotic signals through the mechanosensitive channel Piezo1. In load-bearing cartilage from patients with OA, typical ferroptosis-related mitochondrial morphological changes have been observed; mechanistic studies have shown that excessive mechanical stress activates Piezo1, leading to Ca²^+^ influx, downregulation of GPX4, and enhanced endoplasmic reticulum stress, thereby promoting ferroptosis and accelerating cartilage degeneration (45, 110–112). In conditional GPX4 knockout mice, cartilage-specific loss of GPX4 markedly aggravates experimental OA, whereas supplementation with ferroptosis suppressor protein 1 (FSP1) and coenzyme Q10 (CoQ10) can alleviate the associated damage to some extent, suggesting that GPX4-independent compensatory anti-ferroptotic pathways may also exist in chondrocytes (45). Beyond senescence and ferroptosis, chondrocytes are also co-regulated by hypoxia-related and metabolic reprogramming signals. Existing studies have shown that HIF-1α and hypoxia-inducible factor-2α (HIF-2α) do not play identical roles in chondrocyte ferroptosis: the former may enhance cell survival through metabolic adaptation, whereas the latter appears more likely to promote ferroptosis sensitivity (113, 114). This suggests that, within inflammatory and senescence-related microenvironments, different HIF isoforms may jointly influence chondrocyte fate by regulating energy metabolism, redox homeostasis, and antioxidant defenses. Meanwhile, senescence-related molecules such as microRNA-24 (miR-24) have also been found to correlate with the severity of OA cartilage injury and may influence chondrocyte senescence and repair capacity by regulating targets such as thousand-and-one amino acid kinase 1 (TAOK1) (115). Compared with the direct evidence on ferroptosis and oxidative stress, however, the integrated relationships among these metabolic and transcriptional regulatory mechanisms in OA still require further validation. Overall, chondrocytes in the osteoimmune microenvironment of OA are not only the major recipients of ferroptosis- and senescence-related injury, but also critical hubs that can actively participate in inflammatory amplification through secretion of mediators, matrix remodeling, and cross-tissue communication. However, current understanding remains incomplete as to whether senescent chondrocytes are intrinsically more prone to ferroptosis or instead acquire anti-ferroptotic advantages under specific conditions. This complexity further suggests that future ferroptosis-based interventions should not be simply equated with enhancing cell death, but should instead give greater consideration to precise distinctions in cell state, disease stage, and tissue specificity.

### 4.2 Subchondral bone cells

Accumulating evidence suggests that bone marrow lesions, aberrant angiogenesis, and nerve invasion often emerge before or accompany cartilage degeneration, and by altering mechanical load distribution, releasing bone matrix-derived factors, and opening a vascular–immune “entry point,” they drive local inflammation toward chronicity and self-maintenance (116). In this process, the bone remodeling unit composed of osteoclasts, osteoblasts, and osteocytes no longer merely “repairs microdamage,” but is instead co-opted by mechanical overload, SASP, and disrupted iron homeostasis. On the one hand, abnormal bone remodeling increases vascular permeability, leading to local iron deposition and oxidative stress; on the other hand, ferroptosis in turn further reinforces vascular leakage, inflammatory infiltration, and pain-related pathways, thereby forming a vicious cycle that parallels cartilage degeneration (117). Meanwhile, senescence-associated chronic low-grade inflammation, or inflammaging, provides a sustained pro-inflammatory background within the bone marrow niche, and together these processes shape an osteoimmune microenvironment that is more permissive for OA progression (118).

In early OA, subchondral bone commonly exhibits increased osteoclastic activity and enhanced bone resorption, and only later gradually shifts toward sclerosis and aberrant bone formation (119). Enhanced osteoclastic activity not only alters trabecular architecture, but more importantly releases growth factors embedded in the bone matrix and remodels the local microenvironment, thereby driving angiogenesis and bone marrow lesion formation and creating a “passage” for immune cell entry into the joint organ (120). The significance of the osteoclast lineage in early OA extends beyond increased bone resorption. Osteocyte-derived receptor activator of nuclear factor kappa-B ligand (RANKL)–driven osteoclastogenesis is closely associated with sensory nerve invasion in subchondral bone; netrin-1 secreted by osteoclasts can promote sensory axon growth and mediate OA pain, whereas interfering with osteoclast activity or blocking the netrin-1–deleted in colorectal cancer (DCC) pathway can markedly alleviate nerve invasion and pain-related behaviors (121). In DMM models, preosteoclasts are activated at very early stages and secrete excess platelet-derived growth factor-BB (PDGF-BB), which drives aberrant subchondral angiogenesis through platelet-derived growth factor receptor-β (PDGFR-β) signaling and is coupled with perivascular nerve invasion, structural damage, and pain; knockout or overexpression of PDGF-BB can respectively attenuate or reproduce this pathological vascular–osteogenic cascade (122). Excessive differentiation of osteoclasts in subchondral bone marrow appears to be ferroptosis-dependent and is driven by senescence-associated SASP. Modulation of the DNA damage response-related p53–forkhead box O3 (FOXO3) axis or intra-articular delivery of FOXO3 can simultaneously improve cartilage degeneration and abnormal subchondral bone remodeling (107). As the “command system” for mechanosensation and remodeling, osteocytes can disrupt subchondral bone homeostasis through matrix metalloproteinase-13 (MMP13)-dependent mechanisms and promote OA progression; at the same time, osteocyte-derived RANKL can drive osteoclastogenesis upstream, thereby translating mechanical stress into persistent bone remodeling and pain amplification (123). Li et al. proposed the concept of a “senescent skeletal unit” in OA subchondral bone, in which epiregulin-positive (EREG^+^) macrophages promote senescence and aberrant osteogenesis of epidermal growth factor receptor-positive (EGFR^+^) mesenchymal descendant stem/progenitor cells (MDSPCs) through EREG–EGFR signaling; adeno-associated virus (AAV)-mediated knockdown of Ereg alleviated subchondral sclerosis and reduced pain (124). Based on paired single-cell RNA sequencing of bone marrow lesion (BML) and non-BML regions, Luo et al. found that non-classical monocytes in BML areas were more pro-inflammatory and had higher senescence scores; cell–cell communication analysis suggested that tumor necrosis factor (TNF) signaling drives cartilage damage, and they proposed transcription factor 7-like 2 (TCF7L2) as a shared senescence hub in monocytes and chondrocytes that promotes SASP amplification of bone marrow–cartilage crosstalk (125). In subchondral bone endothelial cells, increased ras homolog family member A (RhoA) can induce endothelial ferroptosis and weaken intercellular junctions, resulting in increased vascular permeability; inhibition of RhoA can alleviate ferroptosis as well as cartilage degeneration and subchondral remodeling in DMM models (117). These findings suggest that ferroptosis in subchondral bone does not occur only in bone cells themselves; vascular endothelium also serves as a critical gatekeeper. Once permeability increases, microbleeding, iron deposition, infiltration of inflammatory mediators, and immigration of immune cells become more likely, further pushing the joint microenvironment toward inflammaging.

### 4.3 T cells

Within the osteoimmune microenvironment of OA, abnormal infiltration and functional skewing of multiple T-cell subsets can be detected in synovial tissue and synovial fluid, involving T helper 1/T helper 17 (Th1/Th17) cells, cytotoxic T cells, and memory T cells. Some studies have suggested that the degree of synovial T helper cell infiltration is associated with clinical symptoms and functional limitation, while earlier studies also observed biased TCR repertoires and clonal expansion in OA synovium, suggesting that T cells may participate in chronic inflammatory circuits driven by persistent antigen stimulation or danger signals (126). Meanwhile, common phenotypes of immunosenescence, such as terminal T-cell differentiation and reduced CD28 expression, have also been reported in the peripheral blood and synovial compartment of patients with OA. These “senescence-like” T cells are more likely to be accompanied by elevated inflammatory mediators and impaired immune surveillance, thereby providing a basis for the long-term maintenance of low-grade intra-articular inflammation (127, 128). In invariant natural killer T (iNKT) cells, GPX4 deficiency leads to increased lipid peroxidation, accumulation of mitochondrial ROS, and impaired cytokine production, including reduced IFN-γ in the NKT1 subset (129). This results in reduced iNKT-cell numbers and functional impairment, as shown in T-cell-specific Gpx4 knockout mouse models, in which ferroptosis inhibitors such as vitamin E or iron chelators can rescue these defects. These findings highlight ferroptosis as a regulator of T-cell survival and effector function, particularly under oxidative stress conditions that are common in aging (71). The local OA environment itself provides conditions that promote both lipid peroxidation and iron accumulation. On the one hand, the association between abnormal iron metabolism and OA has been repeatedly reported in clinical studies, including elevated serum ferritin in patients with OA and its correlation with symptoms and imaging progression, as well as evidence of increased synovial fluid ferritin levels in OA under specific genetic backgrounds such as HFE mutations (130). These changes create an iron-rich and pro-oxidative background within the joint cavity. On the other hand, elevated levels of lipid peroxidation products, particularly 4-hydroxynonenal (4-HNE), have been reported in OA synovial cells and synovial fluid and are thought to directly participate in cartilage matrix degradation and inflammatory amplification (131). Accordingly, T cells in the joint cavity are likely to be chronically exposed to the dual pressure of oxidized lipids and iron excess, and their capacity to counter oxidative stress and clear lipid peroxides may represent a key threshold determining their survival and effector function. There may also be more upstream coupling points between immunosenescence and ferroptosis. Recent studies have shown that methyltransferase-like 3 (METTL3), an RNA methyltransferase, maintains GPX4 expression at the translational level in thymocytes. When METTL3 is conditionally deleted in T cells or thymocytes, double-positive (CD4^+^CD8^+^) thymocytes become more susceptible to ferroptosis and display an accelerated thymic involution phenotype, whereas pharmacological inhibition of ferroptosis improves cell survival and alleviates these “aging-like” changes (132). It should be emphasized that direct causal evidence linking the “iron-rich and oxidized lipid-rich joint cavity” in OA to “increased susceptibility of T cells to ferroptosis” remains limited. However, other disease contexts have provided transferable mechanistic templates. For example, in the tumor microenvironment, cluster of differentiation 36 (CD36)-mediated uptake of oxidized lipids can trigger accumulation of lipid peroxidation in CD8^+^ T cells and activate p38 signaling, leading to reduced effector function; inhibiting p38 or enhancing GPX4-dependent clearance of lipid peroxides can restore the function of tumor-infiltrating CD8^+^ T cells *in vivo* (72). The OA joint cavity may similarly drive T-cell functional exhaustion or survival selection through oxidized lipid stress, thereby reshaping local immune homeostasis. On the other hand, certain T-cell subsets possess a metabolic advantage in resisting ferroptosis. T cytotoxic 9 (Tc9) cells can drive FAO through the IL-9/STAT3 pathway and reduce lipid peroxidation, thereby resisting ROS-induced tumor-associated ferroptotic stress and acquiring more durable survival and effector function (74). In B-cell lymphoma models, lymphoma exposure induces transcriptional and epigenetic changes in young T cells that mimic aged T cells, including upregulation of iron homeostasis genes and resistance to ferroptosis. This resistance is associated with proteostasis defects and accompanied by increased expression of senescence markers such as cyclin dependent kinase inhibitor 2A (Cdkn2a) and tumor necrosis factor alpha (Tnfa). Lymphoma-exposed T cells accumulate iron and resist ferroptosis, mirroring aged T cells and highlighting how chronic disease may accelerate T-cell senescence through ferroptosis-related regulation (133). For OA, these findings provide a mechanistic rationale for future investigation rather than direct therapeutic evidence. Future studies should determine whether OA-infiltrating T cells indeed experience sufficient iron and oxidized lipid stress to undergo ferroptosis-related dysfunction.

### 4.4 Synovial macrophages

**Figure 4:**
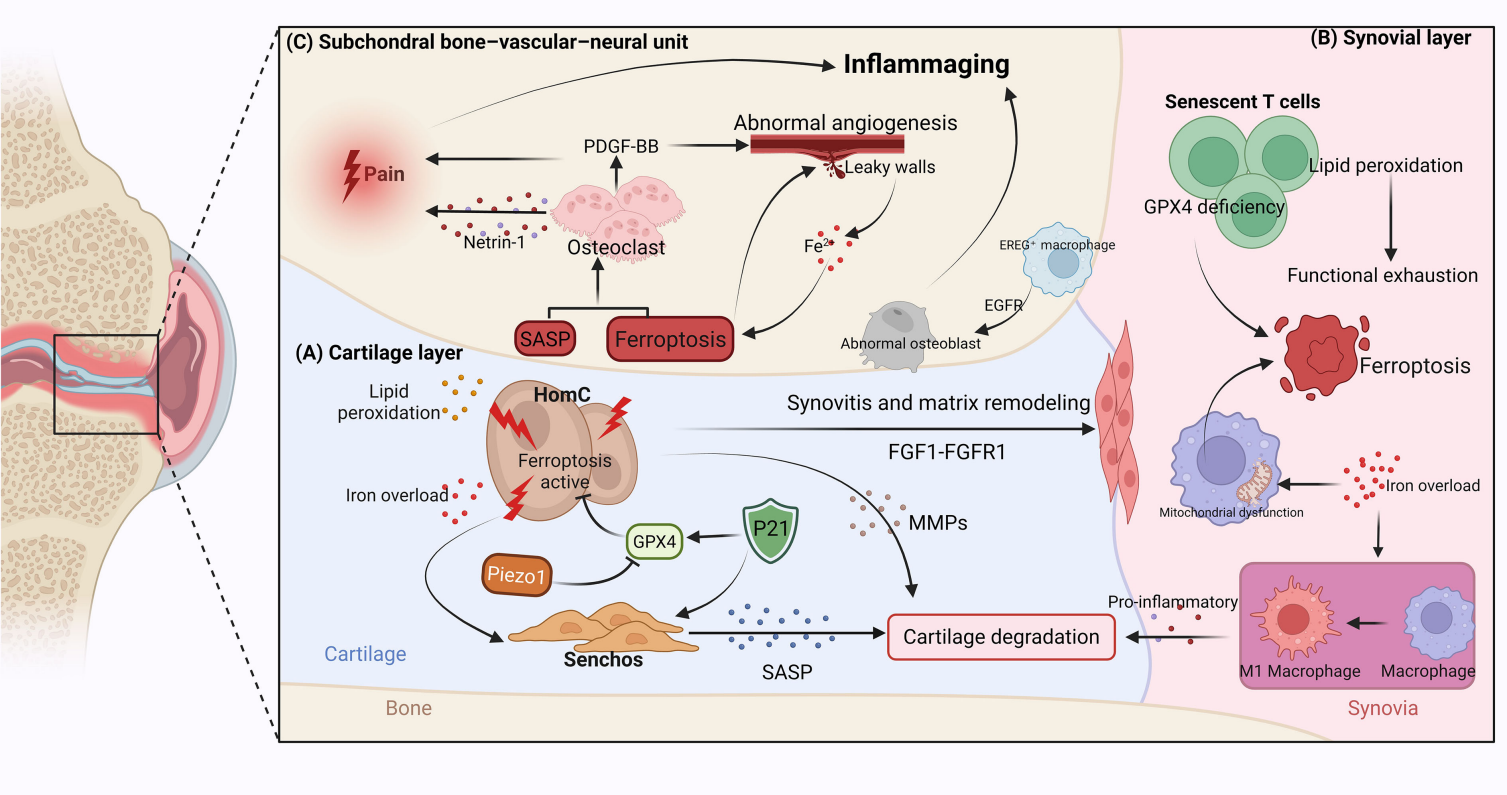
Cellular hubs and inter-tissue crosstalk driving inflammaging and ferroptosis sensitization across cartilage, synovium, and the subchondral bone–vascular–neural unit in osteoarthritis.

Within the osteoimmune microenvironment, synovial macrophages are among the earliest and most direct cell populations to encounter blood degradation products and hemosiderin within the joint cavity, making them the first checkpoint linking iron metabolism dysregulation to immune activation (59). In the proposed iron–senescence framework, macrophages may function not only as targets of iron-related stress but also as amplifiers of inflammatory signaling. In knee OA models, intracellular iron overload promotes M1-like synovial macrophage polarization through the mTORC1-p70S6K/4E-BP1 pathway and aggravates cartilage injury (19). These findings identify the iron burden–myeloid inflammatory shift as a potential local intervention node. At the same time, expression of iron-regulatory molecules can be detected in human synovial tissue, indicating the presence of a local iron flux regulatory platform within the joint that can be remodeled by inflammation and, under persistent synovitis, may favor intracellular iron retention and oxidative pressure in inflamed synovium (134). Evidence from iron-rich arthritic contexts suggests that M2-like macrophages may be more susceptible to ferroptosis than M1-like macrophages, although this remains to be verified in OA synovium. Mechanistically, ferroptotic M2 macrophages release DAMPs such as HMGB1, which binds to TLR4 on M1 macrophages, activates downstream STAT3 signaling, and promotes the production of M1-associated pro-inflammatory cytokines, thereby forming a positive inflammatory feedback loop (63). This observation suggests that anti-inflammatory M2 macrophages, while attempting to repair tissue, are more likely to die under iron overload, whereas pro-inflammatory M1 macrophages survive more effectively in the same iron-rich environment. If present in OA, such selective vulnerability could further contribute to M1/M2 imbalance and persistent synovial inflammation. Senescence further magnifies the destructive role of macrophages in joint pathology. A key feature of senescent macrophage reprogramming is impaired efferocytosis. In late-stage knee OA, impaired efferocytosis of synovial macrophages leads to the accumulation of apoptotic cells and compromises a key homeostatic function of the synovium. Although this mechanism has been described mainly in iron-rich arthritic or non-OA inflammatory contexts, whether selective ferroptosis of M2-like macrophages occurs in OA synovium remains to be directly verified. Accumulated ferroptotic debris may releases DAMPs such as HMGB1, activates TLR4 and RAGE, promotes NLRP3 inflammasome activation, and induces IL-1β/IL-18 release, thereby forming an inflammatory cycle (135, 136). In obesity-associated OA, enhanced M1 polarization leads to downregulation of GAS6, weakens synovial macrophage efferocytosis of apoptotic cells, and promotes synovial hyperplasia and joint degeneration (137). In OA synovium, senescent macrophages exhibit enhanced M1 polarization, mitochondrial damage, and impaired phagocytic capacity, all of which contribute to increased SASP within the joint. Interventions targeting senescent macrophages through the STAT3/ADAM17/MerTK pathway can alleviate trauma- and aging-driven OA phenotypes, causally highlighting that immunosenescence of synovial macrophages is not merely a bystander phenomenon but an actionable driver (57). The consequences of macrophage polarization extend beyond stronger inflammation and may directly alter chondrocyte fate. Studies have shown that promotion of M1 polarization in synovial macrophages aggravates OA progression by secreting R-spondin-2 and activating β-catenin signaling in chondrocytes (138). Evidence also indicates that OA-FLS exosomes deliver miR-19b-3p, which enhances ROS, Fe²^+^, and lipid peroxidation-related indices and promotes chondrocyte ferroptosis by targeting and suppressing SLC7A11, ultimately exacerbating cartilage injury (139). More recent studies further suggest that the metabolic state of synovial macrophages, such as enhanced glycolysis, is closely associated with ferroptotic phenotypes in chondrocytes. Increased glycolysis in synovial macrophages leads to lactate accumulation and lactylation of CD11b at the K575 site. This post-translational modification alters the conformation of CD11b, impairs macrophage recognition of apoptotic cells, compromises synovial macrophage phagocytic capacity, and thereby intensifies inflammation and accelerates OA progression (140). Even more striking is the remote effect of macrophages on periarticular muscle. A pioneering study found that senescent macrophages in the quadriceps of OA mice suppress the mTORC1-HMGCR pathway in muscle cells through paracrine factors, leading to reduced CoQ10 synthesis, impaired clearance of lipid peroxides, and ultimately ferroptosis and muscle atrophy in skeletal muscle cells (82). This finding reveals that senescent immune cells may remotely induce ferroptosis in other tissues, forming a pathological loop of “joint inflammation–macrophage senescence–muscle ferroptosis–functional decline,” thereby aggravating joint mechanical loading and perpetuating disease progression. Overall, through maintenance of barrier homeostasis and efferocytosis, immunosenescence-associated functional decline, iron burden-driven pro-inflammatory polarization, and cross-tissue regulation of cartilage and subchondral bone marrow immune ecology, synovial macrophages constitute a central hub in local osteoimmune microenvironment imbalance and heightened ferroptosis susceptibility in OA (**Figure 4**).

## 5 Preclinical and early translational exploration of interventions targeting ferroptosis- and immunosenescence-related processes

Before discussing therapeutic strategies, it is important to emphasize that most interventions targeting the ferroptosis–immunosenescence connection in OA remain at a preclinical stage. Evidence for many approaches is derived from *in vitro* systems, chemically or surgically induced OA animal models, aging-related disease models, or proof-of-concept studies. Therefore, these strategies should be interpreted as mechanism-oriented therapeutic opportunities rather than clinically validated interventions. Their translational potential will depend on several unresolved factors, including disease-stage specificity, cell type selectivity, intra-articular retention, dosing frequency, long-term safety, off-target effects, biomaterial reproducibility, and validation in large-animal models and clinical studies.

### 5.1 Senolytics and ferroptosis

Traditional senolytics generally eliminate senescent cells by inducing apoptosis. However, increasing evidence in recent years suggests that ferroptosis may also serve as an execution mechanism for senolysis. The BET inhibitor JQ1 has been found to selectively clear senescent cells. Mechanistically, because senescent cells are often characterized by increased ferritin levels, JQ1 specifically triggers ferroptosis in senescent cells by downregulating GPX4 and SLC7A11 while upregulating p53 in these cells (141). Several experimental senolytic strategies have been reported to involve ferroptosis-related mechanisms, although most of these findings currently derive from non-OA models. For example, lipid-based senolytics identified through screening appear to act mainly on ferroptosis-related lipid remodeling and oxidative pathways, such as ACSL4, LPCAT3, and ALOX15, thereby promoting lipid peroxidation and ferroptosis-related senescent cell clearance in experimental settings (142). Feng et al. proposed that senescent cells exhibit lysosomal iron retention, which limits free iron and the labile iron pool (LIP), thereby conferring intrinsic resistance to ferroptosis; when lysosomal function is disrupted using chloroquine or LLOMe, senescent cell death increases significantly (143). In contrast, actively enhancing ferritinophagy to increase the LIP and trigger ferroptosis represents an opposite strategy. For example, the flavonoid compound 4,4′-dimethoxychalcone (DMC) can inhibit FECH and activate ferritinophagy, thereby increasing the LIP and inducing ferroptosis in senescent cells. Moreover, DMC combined with dasatinib and quercetin shows higher senescent cell clearance efficiency, suggesting that ferroptosis sensitization may complement traditional senolytics, although OA-specific validation is still needed (144). Ferroptosis is considered to participate in chondrocyte injury and degeneration; however, some senescent chondrocytes (SenChos) exhibit ferroptosis resistance and become a source of SASP, making them candidates for selective elimination. Wen et al. proposed that SenChos possess a key anti-ferroptotic axis, EAAT1-Glu-GSH-GPX4, and showed that intra-articular injection of the EAAT1 inhibitor UCPH-101 selectively eliminated SenChos, improved cartilage homeostasis, and delayed OA progression (104), thereby providing OA-related preclinical evidence that senolysis may be achieved by targeting ferroptosis resistance in senescent chondrocytes. Nevertheless, whether this approach can be generalized across OA stages, species, and different senescent cell states remains unclear. At the same time, Zheng et al. reported that p21 is highly expressed in OA cartilage and exerts anti-ferroptotic effects by regulating GPX4 protein stability; p21 deficiency renders chondrocytes more sensitive to ferroptosis and aggravates cartilage degeneration, suggesting that simply enhancing ferroptosis may carry the risk of collateral cartilage injury (105). A more precise senolytic design strategy has also been proposed based on a magnetic nanoparticle platform targeting the senescent cell surface marker CD26. In combination with the HSP90 inhibitor 17-DMAG, this platform induces apoptosis together with ferroptosis-related mechanisms, thereby specifically targeting and eliminating senescent cells (145). In addition, senolytic strategies targeting mitochondrial integrity have also been explored. For example, tamoxifen derivatives targeted to mitochondria can selectively eliminate senescent cells by inhibiting oxidative phosphorylation and respiratory chain complex IV activity, thereby making ferroptosis the dominant mode of cell death (146). Meanwhile, intracellular protective factors may also affect clearance efficiency. For instance, in therapy-induced senescent lung cancer cells, cytoplasmic p21 has been considered protective against both senolysis and ferroptosis, suggesting that p21 status may influence the therapeutic window and tolerance assessment for senescent cell clearance (147). Although ferroptosis-based senolytics provide an attractive conceptual link between senescence clearance and ferroptosis regulation, most current evidence is derived from non-OA or proof-of-concept models. Therefore, whether this strategy can selectively eliminate senescent OA cells without damaging non-senescent chondrocytes, synovial cells, or subchondral bone cells remains to be carefully evaluated.

### 5.2 Iron chelators and antioxidants

Increasing evidence suggests that anti-ferroptotic axes such as GPX4 are impaired in OA cartilage, while dysregulated iron homeostasis and lipid peroxidation jointly drive chondrocyte death and matrix degradation; therefore, strategies targeting iron control and antioxidation have clear mechanistic rationale, but their translational value in OA still requires further validation (12, 148). However, these strategies should not be interpreted as direct validation of a ferroptosis–immunosenescence causal loop, because most interventions simultaneously affect iron metabolism, oxidative stress, inflammation, and senescence-related markers. Deferoxamine (DFO) is a classical iron chelator that effectively reduces the intracellular iron pool and inhibits the Fenton reaction. Animal studies have demonstrated that DFO exerts multi-level protective effects within the joint cavity. At the level of iron metabolism, DFO blocks ferritinophagy by inhibiting NCOA4 expression, restores intracellular ferritin storage function, and directly chelates excess free iron, thereby re-establishing iron homeostasis. At the level of oxidative stress, DFO activates the NRF2 antioxidant master pathway, enhances chondrocyte resistance to lipid peroxidation, and thereby suppresses ferroptosis. At the level of tissue remodeling, intra-articular injection of DFO significantly promotes the synthesis and deposition of type II collagen, while downregulating the expression of cell-cycle inhibitors P16 and P21 as well as the matrix-degrading enzyme MMP13, thereby delaying chondrocyte senescence and extracellular matrix degradation and ultimately slowing OA progression (149, 150). In addition to local administration, Burton et al. found in primary/age-related OA models that systemic DFO administration reduced iron burden across multiple tissues, lessened knee cartilage lesions, and improved mobility-related indicators, suggesting that iron chelation delayed the progression of primary OA in animal models and may provide a rationale for further translational evaluation (151). However, the clinical translation of iron chelation still faces practical bottlenecks. Traditional FDA-approved iron chelators such as DFO have limitations including suboptimal pharmacokinetics, non-specific tissue distribution, and adverse effects. Therefore, more feasible approaches often involve intra-articular local administration or the use of biomaterial-based systems to achieve retention and controlled release, thereby minimizing systemic exposure (152). The key oxidative damage in OA is often not ROS in a general sense, but rather PUFA-PL lipid peroxidation chain reactions tightly coupled to ferroptosis, together with mitochondrial oxidative sources associated with mechanical and inflammatory stimuli (8, 9, 31). N-acetylcysteine (NAC), as a precursor for GSH synthesis, can enhance cellular antioxidant capacity and function as an antioxidant by targeting ROS. Animal studies have shown that oral NAC can attenuate OA-related changes in anterior cruciate ligament transection (ACLT) and related models (153). The mitochondria-targeted antioxidant MitoQ has been reported to alleviate oxidative stress and improve pathological progression in OA models through activation of the NRF2/Parkin axis, thereby delaying OA progression (154). Among natural antioxidant molecules, astaxanthin has been reported to alleviate OA progression by inhibiting ferroptosis and modulating mitochondrial function (155). Multiple studies have also supported the anti-ferroptotic and chondroprotective effects of melatonin from the perspectives of the NOX4–mitochondrial oxidative stress axis and the SLC7A11/GPX4 axis (156, 157). Icariin (ICA) can activate the SIRT1–NRF2–HO-1 antioxidant axis in OA models, improve cartilage pathology, and reduce apoptosis and DNA damage caused by inflammatory stimulation *in vitro* (158). It can also inhibit chondrocyte ferroptosis and alleviate OA progression by enhancing SLC7A11/GPX4 signaling (159). Rutin can activate SIRT1 in chondrocyte oxidative stress models, suppress NF-κB/MAPK inflammatory signaling, and reduce inflammatory cytokine expression (160). Collectively, these studies are pushing antioxidant strategies in OA beyond general free radical scavenging toward targeted inhibition of lipid peroxidation and mitochondrial oxidative sources, thereby suppressing the sustained generation of ferroptotic and senescent phenotypes.

### 5.3 Metabolic reprogramming

The OA joint is not merely a site of mechanical wear, but rather a microenvironment chronically exposed to hypoxia, inflammation, and metabolic stress. Declining mitochondrial function, NAD^+^ depletion, and energy metabolism imbalance in chondrocytes can trigger ROS accumulation and lipid peroxidation, thereby promoting ferroptosis and the DNA damage response; the latter further activates senescence pathways such as p53–p21 and p16–Rb and amplifies SASP (161, 162). Reduced AMPK activity is considered one of the key features of disrupted metabolic homeostasis in OA chondrocytes. Activation of AMPK can suppress inflammation-induced catabolic responses and promote autophagy, thereby counteracting the accumulation of cellular stress and senescent phenotypes (163). On this basis, metformin is one of the most promising metabolic drugs. Studies have shown that metformin can alleviate OA cartilage degeneration and reduce cartilage aging and senescence-associated changes through the AMPK/mTOR pathway, suggesting that its effects are not limited to anti-inflammation, but also involve regulation of the “metabolism–senescence axis” (164). Consistent with the effects of metformin, direct activation of AMPK can also inhibit chondrocyte senescence. In chondrocyte models exposed to high-glucose stress, the AMPK agonist AICAR promotes autophagy while simultaneously suppressing apoptosis and senescence-associated alterations (165). Animal studies have shown that intra-articular injection of rapamycin can delay OA-like changes. Further delivery optimization, such as rapamycin-loaded microparticles or hydrogels, not only prolongs joint retention time but also shows potential in experimental models to suppress senescence, reduce intra-articular senescence burden, and improve OA (166). Increasing evidence suggests that OA cartilage exhibits reduced NAD^+^ levels and disruption of related metabolic networks, and that NAD^+^ deficiency mechanistically contributes to disease initiation and progression. At the cellular level, supplementation with the NAD^+^ precursor nicotinamide riboside can increase intracellular NAD^+^ levels in OA chondrocytes and counteract inflammation-induced NAD^+^ decline and matrix catabolic changes, indicating that NAD^+^ replenishment has a clear metabolic repair rationale (162). Some studies have linked NAD^+^ precursor delivery with the local joint microenvironment and used material-based systems to reactivate mitochondrial function, thereby suppressing SASP, preserving cartilage integrity, alleviating chondrocyte senescence, and preventing OA progression (167). OA synovitis and macrophage activation also display marked metabolic phenotypes, with pro-inflammatory states often accompanied by enhanced glycolysis and amplification of specific metabolite-derived signals. In this context, the itaconate pathway has emerged as a notable immunometabolic target in recent years. Studies have shown that 4-octyl itaconate can suppress synovitis and improve pain in post-traumatic OA (PTOA) models and is associated with regulation of inflammatory phenotypes in macrophages and synovial tissue. Other studies suggest that 4-octyl itaconate can enhance autophagy in chondrocytes and improve OA by inhibiting the PI3K/AKT/mTOR pathway (168, 169). Another example is dimethyl fumarate (DMF). Recent studies suggest that DMF alleviates OA progression by activating NRF2, reducing ROS, suppressing osteoclastogenesis in early OA, preserving the microstructure of subchondral bone, and mitigating articular cartilage damage (170). By reducing the metabolic drivers of synovitis and thereby lessening the secondary impact of inflammatory mediators and SASP on the osteoimmune microenvironment, these findings highlight the therapeutic potential of metabolism-derived agents. Metabolic reprogramming approaches should also be interpreted cautiously, because pathways such as glycolysis, fatty acid oxidation, mitochondrial metabolism, and redox regulation are widely shared across multiple joint-resident and immune cell populations. Future studies should determine whether these interventions act specifically through ferroptosis–immunosenescence interactions or primarily through broader anti-inflammatory, antioxidant, or tissue-protective mechanisms.

### 5.4 Nanomedicine and exosomes: from local delivery to microenvironment remodeling

**Figure 5:**
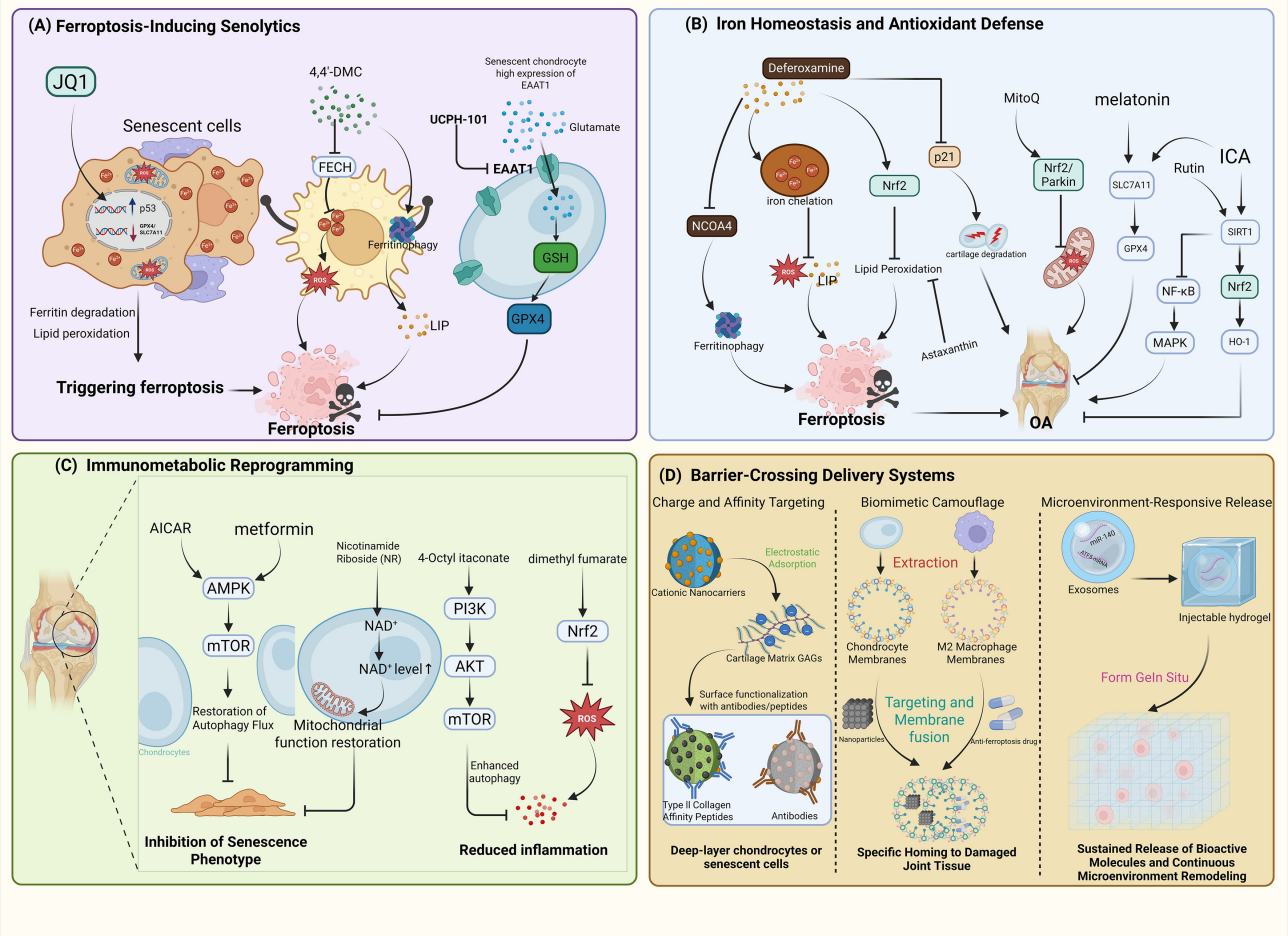
Translational intervention strategies targeting the ferroptosis–immunosenescence connection in OA. (A) Ferroptosis-inducing senolytics. (B) Iron homeostasis and antioxidant defense. (C) Immunometabolic reprogramming. (D) Barrier-crossing delivery systems for local delivery and microenvironment remodeling.

Within the framework of the vicious cycle linking iron overload, oxidative stress, and senescence/inflammation, many candidate interventions, including iron chelators, antioxidants, ferroptosis inhibitors, and metabolic reprogramming agents, face translational bottlenecks such as rapid clearance from the joint cavity, poor penetration through the cartilage barrier, and difficulty in maintaining effective local concentrations. Articular cartilage is dense, avascular, and rich in negatively charged glycosaminoglycans (GAGs), making it difficult for most drugs to reach deep-layer chondrocytes. At the same time, intra-articularly injected drugs are often rapidly cleared by the synovium, resulting in very short retention times (171, 172). Therefore, nanodelivery systems and exosomes are gradually evolving from tools for optimizing drug administration into a toolbox for microenvironment remodeling. The porosity and fixed negative charge of articular cartilage define two core engineering strategies. One is to use cationic nanocarriers that reversibly bind to GAGs, thereby enhancing penetration and retention through electrostatic interactions and improving deep tissue delivery efficiency. The other is to decorate carrier surfaces with antibodies or peptides that have affinity for type II collagen and chondrocytes, thereby increasing tissue selectivity and reducing off-target exposure. Only by delivering molecular interventions into cartilage and the synovium–macrophage niche in a stable, sufficient, and durable manner is it possible to truly interrupt this vicious cycle (171). In addition, some studies have used biomimetic membrane-coated nanoparticles to improve joint retention and enhance cell recognition. Deng et al. improved intra-articular retention and cartilage protection by coating nanoparticles with chondrocyte membranes, demonstrating in rat and canine OA models the feasibility of using chondrocyte membrane-coated nanoparticles to improve the pharmacokinetics and therapeutic efficacy of anti-OA drugs (173). In the study by Zhou et al., an anti-inflammatory system termed M2H@RPK was developed, in which KAFAK and shRNA-LEPR were condensed with polyethylenimine (PEI) to form a complex and subsequently modified with hyaluronic acid (HA) to mask the positive charge of the M2 membrane. This system significantly reduced pro-inflammatory cytokines and synovial inflammation, and exerted notable therapeutic effects in OA by reducing joint damage (174). These findings suggest that activated macrophage membrane-coated nanoparticles may also serve as carriers for therapeutic delivery and immune microenvironment remodeling. Cao and colleagues developed an innovative nanoparticle platform, a bioengineered chondrocyte membrane-camouflaged anti-ferroptotic drug delivery system designed to enhance cartilage penetration, prolong drug retention, and achieve targeted therapy. By constructing a chondrocyte membrane-disguised anti-ferroptotic drug platform, they achieved efficient cartilage-targeted drug delivery for OA treatment (175). Stimuli-responsive hydrogels can form gels *in situ* within the joint cavity and enable controlled release. Thermosensitive, pH-responsive, enzyme-responsive, and multi-responsive hydrogels have become important OA delivery platforms because of their potential for intelligent drug delivery, inhibition of cartilage degradation, and reduction of inflammation (176). Nanomedicine platforms may improve local delivery and intra-articular retention of anti-ferroptotic or anti-senescent agents, but their clinical translation requires careful evaluation of degradation behavior, immunogenicity, local and systemic toxicity, manufacturing reproducibility, sterilization procedures, regulatory requirements, and efficacy in clinically relevant large-animal models. For exosomes, rapid clearance and insufficient retention remain major barriers to clinical translation, and hydrogels are considered an effective strategy to address these issues. For example, exosome–thermosensitive hydrogels can provide sustained release. By *in situ* crosslinking of Pluronic F-127 and HA, a thermosensitive injectable hydrogel was developed as a sustained-release carrier that enables prolonged retention of primary chondrocyte-derived exosomes at sites of cartilage injury, effectively enhancing cartilage matrix formation and promoting macrophage polarization toward the M2 phenotype, thereby alleviating OA (177). This reflects the dual advantages of improved delivery and immune microenvironment remodeling. Small extracellular vesicles (sEVs) derived from the infrapatellar fat pad of patients with OA have been reported to promote cartilage degradation and induce senescence-associated phenotypes; inhibition of exosome biogenesis can significantly attenuate sEV-induced cartilage destruction (178). These findings suggest that, at the translational level, exosome-based strategies should not only focus on supplementation, but also consider blockade of pathological extracellular vesicle pathways. Engineered exosomes have also been combined with injectable biomaterials. Ma et al. loaded activating transcription factor 5 (ATF5)-modified messenger RNA (mRNA) into bone marrow mesenchymal stem cell-derived engineered exosomes. By activating the mitochondrial unfolded protein response within the chondrocyte mitochondrial quality control system, these exosomes alleviated IL-1β-induced inflammation and mitochondrial dysfunction, while promoting ECM synthesis and restoring the balance between anabolic and catabolic processes. They further used an injectable thermosensitive hydrogel composed of poly(lactic-co-glycolic acid)-poly(ethylene glycol)-poly(lactic-co-glycolic acid) (PLGA-PEG-PLGA) as a carrier to construct Gel@Ex^modAtf5, thereby achieving sustained release of engineered exosomes, overcoming the limitation of rapid local metabolism and short duration of action, and improving OA phenotypes (179). Liang et al. engineered CAP-Exos by fusing the chondrocyte affinity peptide (CAP) with the exosomal membrane protein lysosome-associated membrane protein 2B (LAMP2B), enabling efficient loading and delivery of miR-140. Compared with unmodified exosomes, CAP-Exos showed stronger intra-articular retention after injection *in vivo* and delivered miR-140 into deep cartilage regions, where they downregulated cartilage degradation-related proteases and alleviated disease progression in a rat OA model (180). Despite the potential advantages of engineered exosomes as delivery vehicles or mediators of intercellular communication, their translational application in OA remains limited by cargo heterogeneity, donor-cell variability, targeting specificity, biodistribution, dosing control, scalable production, storage stability, and long-term safety (**Figure 5**).

Overall, current therapeutic approaches targeting ferroptosis, senescence, inflammation, or local delivery provide useful mechanistic clues for OA intervention, but they should not be regarded as direct clinical validation of a ferroptosis–immunosenescence causal loop. Because many interventions simultaneously affect iron metabolism, oxidative stress, inflammatory signaling, matrix remodeling, and cellular senescence, future studies should determine which effects are specifically mediated through ferroptosis–immunosenescence interactions. Standardized preclinical models, long-term safety assessment, clinically relevant dosing regimens, and well-designed translational studies will be required before these strategies can be considered for routine OA treatment.

### 5.5 Translational challenges and clinical readiness

Although targeting ferroptosis- and immunosenescence-related processes provides a mechanistically attractive direction for OA therapy, several translational barriers remain. Safety is a major concern because ferroptosis, senescence, and immune remodeling also participate in tissue homeostasis, stress adaptation, host defense, and repair. Therefore, excessive or non-selective modulation of these pathways may damage functional chondrocytes, synovial cells, subchondral bone cells, endothelial cells, or resident immune cells. Specificity is another challenge, as OA involves multiple tissues and cell populations, and the same redox, metabolic, or inflammatory pathway may have different effects depending on cell type, disease stage, mechanical loading, and inflammatory context. Delivery efficiency also remains unresolved. Although intra-articular administration, engineered exosomes, hydrogels, nanoparticles, and other local delivery systems may improve retention and controlled release, their clinical use requires further evaluation of tissue penetration, biodistribution, degradation behavior, immunogenicity, dosing frequency, manufacturing reproducibility, sterilization, scalability, and regulatory feasibility. Finally, human validation is still limited. Most available evidence comes from cultured cells, animal models, or proof-of-concept studies, which cannot fully reproduce the chronic, heterogeneous, mechanically loaded, and multimorbid nature of human OA. Future studies should incorporate human OA tissues, patient-derived cells, explant or organoid systems, spatial and single-cell analyses, large-animal models, and well-designed clinical studies to determine whether these strategies provide disease-modifying benefits beyond general anti-inflammatory, antioxidant, or cytoprotective effects.

## 6 Conclusions and future perspectives

Osteoarthritis (OA) is not merely a local degenerative disorder driven by cartilage wear, but rather a whole-joint pathological process involving cartilage, synovium, subchondral bone, and immune components. Recent studies have shown that ferroptosis and immunosenescence each play important roles in OA initiation and progression. The former is closely associated with dysregulated iron homeostasis, lipid peroxidation, and impaired antioxidant defense, thereby promoting local joint cell injury and matrix destruction; the latter continuously disrupts joint homeostasis through chronic low-grade inflammation, immune cell functional remodeling, and reduced tissue repair capacity. Based on current evidence, ferroptosis and immunosenescence may not be isolated processes, but may converge within the osteoimmune microenvironment through oxidative stress, inflammatory amplification, and metabolic disturbance, thereby potentially contributing to OA progression. However, it should be emphasized that direct evidence demonstrating a complete causal loop between ferroptosis and immunosenescence in OA remains limited. Existing studies are still largely concentrated in *in vitro* experiments, animal models, and mechanistic extrapolation from other disease contexts. Within OA tissues, the modes of interaction among different cell types, their relative contributions at different disease stages, and whether this crosstalk has clear implications for patient stratification all remain to be clarified. Therefore, rather than viewing the ferroptosis–immunosenescence relationship as a fully established unified mechanism, it is more appropriate to regard it as a biologically plausible working framework that still requires continued validation.

Future studies may focus on several priorities. First, by integrating single-cell sequencing, spatial transcriptomics, and lineage tracing approaches, it will be important to define the specific roles of chondrocytes, FLS, synovial macrophages, T cells, and subchondral bone-related cells in this crosstalk. Second, through *in vivo* cell type-specific interventions and temporal analyses, future work should determine whether ferroptosis and immunosenescence form a stable bidirectional reinforcing relationship and clarify their dominant roles across different stages of OA. Third, biomarkers reflecting abnormal iron metabolism, lipid peroxidation, and immunosenescence should be identified to support patient stratification and mechanism-oriented therapeutic strategies. Fourth, at the therapeutic level, greater attention should be paid to the indication boundaries and combinational potential of anti-ferroptotic, anti-senescence, and locally optimized delivery strategies, although their clinical translation still depends on more robust mechanistic validation. Overall, integrating the potential link between ferroptosis and immunosenescence from the perspective of the osteoimmune microenvironment may contribute to a more comprehensive understanding of OA as a complex whole-joint disease. Although this field is still evolving, a research path grounded in evidence stratification and mechanistic validation may provide new insights for precision classification and intervention strategy development in OA.
